# Optimization of time domain diffuse correlation spectroscopy parameters for measuring brain blood flow

**DOI:** 10.1117/1.NPh.8.3.035005

**Published:** 2021-08-12

**Authors:** Dibbyan Mazumder, Melissa M. Wu, Nisan Ozana, Davide Tamborini, Maria Angela Franceschini, Stefan A. Carp

**Affiliations:** Harvard Medical School, Massachusetts General Hospital, Optics at Athinoula A. Martinos Center for Biomedical Imaging, Department of Radiology, Charlestown, Massachusetts, United States

**Keywords:** time domain diffuse correlation spectroscopy, cerebral blood flow measurement, optimization, Monte Carlo simulation, instrument response function

## Abstract

**Significance:** Time domain diffuse correlation spectroscopy (TD-DCS) can offer increased sensitivity to cerebral hemodynamics and reduced contamination from extracerebral layers by differentiating photons based on their travel time in tissue. We have developed rigorous simulation and evaluation procedures to determine the optimal time gate parameters for monitoring cerebral perfusion considering instrumentation characteristics and realistic measurement noise.

**Aim:** We simulate TD-DCS cerebral perfusion monitoring performance for different instrument response functions (IRFs) in the presence of realistic experimental noise and evaluate metrics of sensitivity to brain blood flow, signal-to-noise ratio (SNR), and ability to reject the influence of extracerebral blood flow across a variety of time gates to determine optimal operating parameters.

**Approach:** Light propagation was modeled on an MRI-derived human head geometry using Monte Carlo simulations for 765- and 1064-nm excitation wavelengths. We use a virtual probe with a source–detector separation of 1 cm placed in the pre-frontal region. Performance metrics described above were evaluated to determine optimal time gate(s) for different IRFs. Validation of simulation noise estimates was done with experiments conducted on an intralipid-based liquid phantom.

**Results:** We find that TD-DCS performance strongly depends on the system IRF. Among Gaussian pulse shapes, ∼300  ps pulse length appears to offer the best performance, at wide gates (500 ps and larger) with start times 400 and 600 ps after the peak of the TPSF at 765 and 1064 nm, respectively, for a 1-s integration time at photon detection rates seen experimentally (600 kcps at 765 nm and 4 Mcps at 1064 nm).

**Conclusions:** Our work shows that optimal time gates satisfy competing requirements for sufficient sensitivity and sufficient SNR. The achievable performance is further impacted by system IRF with ∼300  ps quasi-Gaussian pulse obtained using electro-optic laser shaping providing the best results.

## 1 Introduction

The brain of a normal human adult has a high energy demand, receiving 10% to 16% of cardiac output under normal cerebral circulation and cardiac function, while weighing only around 2% of the total body mass.^1^ This demand is met through the delivery of oxygen and glucose by means of blood perfusion into the brain. Cerebral autoregulation is a mechanism that assists in maintaining a relatively stable cerebral blood flow (CBF) over a wide range of cerebral perfusion pressures. However, this protective mechanism becomes impaired under abnormal conditions such as cerebral ischemia, traumatic brain injury, and subarachnoid haemorrhage.^2^^,^^3^ Impairment of cerebral autoregulation under any circumstances may lead to both hypoperfusion (inadequate CBF) and hyperperfusion (excess CBF) that can result in damage to the patient’s brain.^4^ Non-invasive monitoring of CBF at the bedside is needed for CBF management and assessment of CA with the goal of maintaining brain health under these circumstances.^5^

However, the traditional CBF measurement techniques, such as MRI-ASL^6^ and XeCT,^7^^,^^8^ are not suitable for bedside monitoring. Transcranial Doppler ultrasound (TCD),^9^^–^^11^ which is compact enough for the bedside, is not widely used because of its high operator dependency due to strict requirements for stable positioning and alignment,^12^^,^^13^ and because some patients have inadequate transtemporal acoustic windows.^14^^–^^16^ Also, TCD can only measure blood flow from large arteries, such as the middle cerebral artery, and not perfusion in micro-vasculature, where the oxygen and nutrient exchange actually occurs. Cerebral oximeters, based on near-infrared spectroscopy, offer a convenient solution for measuring relative cerebral oxygen saturation (${\mathrm{rSO}}_{2}$) as a surrogate for CBF^17^^–^^20^ with the use of miniature optical probes. However, the use of ${\mathrm{rSO}}_{2}$ as surrogate for CBF is limited to certain assumptions^21^ such as constant hematocrit, arterial saturation, and cerebral oxygen metabolism that may not be valid during pathological and surgical conditions.

Diffuse correlation spectroscopy (DCS),^22^^–^^25^ a near-infrared technique that can overcome most of these challenges, is gaining popularity in the neuromonitoring field. A typical DCS setup to measure CBF employs a long coherence-length laser to illuminate a portion of the subject’s forehead. This leads to the formation of a speckle pattern as multiple-scattered photons, after traversing through different trajectories within the illuminated volume of the head, interfere at the surface. The speckle pattern changes due to the movement of scatterers in the illuminated volume. Usually, the fluctuating intensities of a single speckle are recorded using fast photodetectors over time, and the temporal autocorrelation function is computed. The decay rate of the obtained temporal autocorrelation function is proportional to scatterer motion within the illuminated volume of the subject’s head, which is mainly dominated by the movement of red blood cells (RBCs) within the micro-vasculature. This paves the way to assess perfusion in the probed tissues by means of a DCS-derived blood flow index (BFi).^24^

Since non-invasive operation requires the injection and detection of light from the surface of the subject’s head, the recovered BFi values from DCS measurements are susceptible to contamination by hemodynamic processes in the extracerebral layers,^26^^,^^27^ e.g., scalp and skull. To maximize the sensitivity of CBF in the measured BFi, large source–detector (S–D) separations (∼30  mm) are used in traditional continuous wave DCS measurements, as photons with longer path lengths can be detected in these separations. An inherent drawback with this approach is detecting fewer photons at large S–D separations that detrimentally affects the signal-to-noise ratio (SNR) of the measured temporal autocorrelation function. A time-resolved variant of DCS that allows us to select photons with longer path lengths even at shorter separations (∼10  mm), where detected photons are abundant, has been recently proposed under the name time domain DCS^28^^–^^30^ (TD-DCS). TD-DCS enables the selection of photons based on their time-of-flight (ToF). Ideally, TD-DCS allows one to separate out the photons that have traveled longer distances from the rest even at shorter S–D separations. Longer traveling photons are most likely to travel into the deeper tissues than the shorter traveling counterparts; hence, an exclusive consideration of the former while obtaining the BFi can yield higher sensitivity to CBF.

A TD-DCS device uses a coherent pulsed laser as a source and high-resolution time tagging system on the detector side to record the ToF and absolute time of arrival of photons simultaneously.^28^^,^^30^ Ultimately, the autocorrelation function is computed using the absolute arrival times for the photons whose ToF falls within a desired range of values. This ToF range determines the associated depth of the hemodynamic process that influences the obtained BFi the most. To achieve the optimal performance from this time-domain strategy for CBF monitoring, identification of the best ToF range(s), which will henceforth be referred as the time gate(s), is crucial.

The primary objective of this work is to present a framework for the rigorous simulation and evaluation of optimal time gating parameters for monitoring cerebral perfusion while considering instrumentation characteristics and realistic measurement noise. Simulation studies offer convenient means to compare the performance of a wide variety of system instrument response functions (IRF) and time gates.^31^^,^^32^ In this work, we have modeled the propagation of light through a realistic head geometry using Monte Carlo simulations. We considered a measurement geometry typically used for TD-DCS neuromonitoring with a 1-cm S–D separation virtual probe in pre-frontal region.^29^^,^^33^^,^^34^ Then, the detected photons have been assigned to different time gates based on their ToF and the autocorrelation function has been obtained for each of these gates using the photon information. Here, we have also taken in account the error in the ToF that is introduced due to the non-idealities or real IRFs modeled from existing commercially available hardware. We demonstrate an appreciable difference in CBF sensitivities with various combinations of sources and detectors, and conclude that incorporation of the real IRF while estimating the CBF sensitivity for TD-DCS is important, a factor that has not explored in detail in previous studies.^31^^,^^35^ To best link our optimization procedure with actual outcomes expected in practice, we model DCS measurement statistical noise based on realistic experimental conditions, take into account the effects of the finite coherence length of pulsed lasers (also not considered in previous works), and define a combined metric that accounts for the intrinsic CBF sensitivity as well as both the SNR of the measurement and degree of susceptibility to superficial cross-talk. The accuracy of the predicted TD-DCS BFi SNR was further verified against liquid phantom measurements. Based on this simulation approach and combined performance metric, we define the optimal operating conditions for TD-DCS at 765 and 1064 nm taking into account the actual source and detector performance characteristics with the assumed goal of maximizing the ability to detect changes in CBF in the presence of experimental noise and extracerebral interference.

## 2 Methods

### 2.1 Forward Model

#### 2.1.1 Theory

A typical DCS measurement involves computing the normalized intensity autocorrelation function ($g_{2}$) from the time course of scattered light intensity. The recorded $g_{2}$ carries information regarding the speed of the movement of RBC through microvasculature in the illuminated region of the tissue, quantified as BFi.^24^ To simulate $g_{2}$ curves for various experimental conditions, we used detected photon information taken from modeling light propagation through the medium using Monte Carlo, as described next.

In the present work, we have employed the Monte Carlo eXtreme (MCX) package, a parallel Monte Carlo algorithm, accelerated by graphics processing units that can model photon propagation in arbitrary 3D media.^36^ In this algorithm, a given number of simulation threads are launched, where each thread simulates a sequence of photon migration.^36^ Here, photons packets are launched from a source and propagate according to the scattering length ($l^{*}$) and scattering anisotropy (g) of the medium before potentially getting detected at discrete detector positions. For each detected photon packet, the total path length L and total momentum transfer Y in each tissue type, accumulated over all scattering events, are recorded. The momentum transfer for a single scattering event is given by $\overset{\to }{q}={\overset{\to }{k}}_{\text{out}}-{\overset{\to }{k}}_{\text{in}}$, where ${\overset{\to }{k}}_{\text{out}}$ and ${\overset{\to }{k}}_{\text{in}}$ are the photon packet wave vectors that are scattered from the scattering center and incident on the scattering center, respectively. The total dimensionless momentum transfer $Y=\sum q^{2}/2k_{0}^{2}$ is recorded, where the sum is over all scattering events and $k_{0}$ is the wavenumber of incident light. This recorded photon packet specific information has been used to compute the temporal field autocorrelation function $G_{1}(\tau )$ for each layer using:^37^^,^^38^

$$G_{1}(\tau )=\frac{1}{N_{p}}\overset{N_{p}}{\underset{n=1}{\sum }}\exp(\;-\frac{1}{3}k_{0}^{2}\overset{N_{t}}{\underset{i=1}{\sum }}Y_{n,i}{\left\langle \Delta r^{2}(\tau )\right\rangle }_{i})\exp(-\overset{N_{t}}{\underset{i=1}{\sum }}\mu_{a,i}L_{n,i}),$$ (1)

where $N_{p}$ is the number of photon packets detected, $N_{t}$ is the number of tissue layers, $Y_{n,i}$ is the total momentum transfer for photon packet n in layer i, $L_{n,i}$ is the total path length of photon packet n in layer i, $\mu_{a,i}$ is the absorption coefficient in layer i, τ is the delay time, and ${\left\langle \Delta r^{2}(\tau )\right\rangle }_{i}$ is the mean square displacement of the scattering particles in layer i, which is given as ${\left\langle \Delta r^{2}(\tau )\right\rangle }_{i}=6D\tau$, where D is the effective diffusion coefficient, also referred to in the field as the blood flow index or BFi. The normalized temporal field autocorrelation function $g_{1}$ (τ) is obtained as $g_{1}(\tau )=G_{1}(\tau )/G_{1}(\tau =0)$ and finally, $g_{2}$ is obtained using Siegert relation $g_{2}(\tau )=1+\beta {\left|g_{1}(\tau )\right|}^{2}$, where β is the coherence factor.

#### 2.1.2 Monte Carlo simulation scenarios

**Fig. 1:**
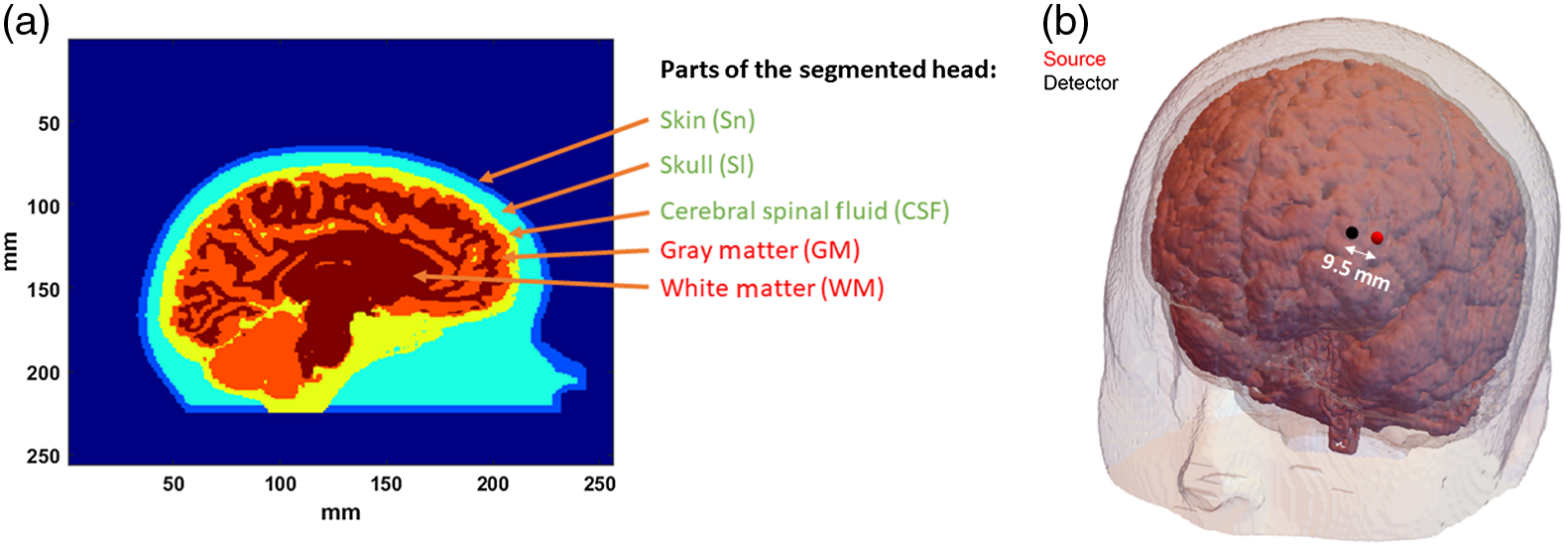
(a) Human head cross section from MRI structural scan, showing different parts of the head. The Sn, Sl, and CSF have been considered extracerebral layers, with GM and WM combined as brain. The dynamics in the Sl and CSF region were considered to be zero. (b) The position of the source and detector locations on the forehead, away from the midline of the brain.

As mentioned earlier, we used MCX to implement the modeling of photon propagation in tissue. In our simulations, we launched 6 billion photon packets into our 3D segmented human head geometry. The segmented volume, whose cross-section has been shown in Fig. 1(a), was obtained from an MRI structural scan of a human subject. The resolution of the geometry was 1  mm×1  mm×1  mm. We have categorized different parts of the head into five layers: (i) skin (Sn), (ii) skull (Sl), (iii) cerebral spinal fluid (CSF), (iv) gray matter (GM), and (v) white matter (WM). To simulate a more challenging condition where the subject has a thicker extracerebral layer, a new geometry was created wherein the thickness of the Sn and Sl layers of the segmented head was incremented outward along the radial direction using 3D image dilation. The thickness of the combined Sn and Sl regions for the new head geometry was approximately 3 mm more than the thickness of combined Sn and Sl regions of the original head model that was used in most of our simulations. The radial distance between the surface of the forehead and the cortex was around 14 mm for the original head model and for the modified head geometry, it was approximately 17 mm.

Given diffusive propagation of light in tissue, we have considered a point source in our simulations. The detector is circular with a diameter of 1 mm. The probe consists of an arrangement of a source and a detector with the distance between the source and the detectors maintained at 10 mm. After wrapping the probe around the segmented head, the new straight-line distance between the source and the detector was 9.5 mm. To reflect the advantage of the time-domain technique over continuous-wave, we do not consider S–D separations longer than 9.5 mm; this also allows for higher photon counts for a given path length distribution by avoiding the geometrical 1/r loss at larger separations. The final arrangement of the probe around the segmented head has been shown in Fig. 1(b). We placed the virtual probe in the prefrontal region, somewhat laterally where bone thickness is generally lower. Our wavelengths of interest for illuminating the human head were 765 and 1064 nm, reflecting currently available choices in pulsed laser sources suitable for TD-DCS.^30^^,^^34^ The optical properties of the five segments of our head model have been reported in Table 1. The reduced scattering coefficients ($\mu_{s}^{\prime }$) of all the segments other than CSF in the table have been obtained from Farina et al.^39^ To determine the $\mu_{s}^{\prime }$ of CSF, we have assumed a power law for dependence of $\mu_{s}^{\prime }$ on wavelength (λ) as $\mu_{s}^{\prime }(\lambda )=0.0016\times \lambda^{-0.44}$. We obtain the 0.0016 multiplicative constant by assuming $\mu_{s}^{\prime }$ to be $0.1\;{\mathrm{cm}}^{-1}$ at 800-nm excitation wavelength as reported in Okada et al.^40^ For obtaining the absorption coefficients ($\mu_{a}$) in Table 1, we have collected the values of the molar extinction coefficients from Refs. 41–45 and have assumed the [HbO] to be 20  μM, [HbR] to be 10  μM, and water content to be 60% for skin layer; whereas for the gray and white matter regions, as [HbO] of 50  μM, [HbR] of 30  μM, and 75% of water content was used. The absorption of the skull layer (Sl) was assumed to be 1.22 times^46^^,^^47^ of skin layer (Sn). The $\mu_{a}$ of the CSF was assumed to be the same as water. For the MC simulations, we computed $\mu_{s}$ assuming g=0.9.^47^^–^^50^ The refractive index for all the layers was assumed to be 1.4.^50^

**Table 1:** Optical properties for different segments of the head geometry.

Tissue type	$\mu_{s}^{\prime }$ in ${\mathrm{cm}}^{-1}$ (765 nm)	$\mu_{s}^{\prime }$ in ${\mathrm{cm}}^{-1}$ (1064 nm)	$\mu_{a}$ in ${\mathrm{cm}}^{-1}$ (765 nm)	$\mu_{a}$ in ${\mathrm{cm}}^{-1}$ (1064 nm)
Sn	9.68	8.37	0.076	0.105
Sl	9.68	8.37	0.093	0.128
CSF	0.1	0.09	0.025	0.122
WM	9.68	8.37	0.188	0.173
GM	9.68	8.37	0.188	0.173

#### 2.1.3 Incorporation of IRF

The photon path lengths sampled from the Monte Carlo simulations correspond to pulse excitation with an infinitely narrow width [a Dirac delta function (δ)-pulse]. To incorporate the effect of the IRF, the tissue temporal point spread function (tissue-TPSF) obtained from these path lengths can be convolved with the system IRF for a given pair of source and detector hardware (S–D pair) to generate the overall temporal point spread function (overall-TPSF).^51^ To do this, we used simulated Gaussian IRFs, or obtained the IRF experimentally from the histogram of the elapsed time between laser pulses and reported photon detections when the source and detector fibers directly faced each other. The histogram was then normalized to obtain the corresponding probability density function (PDF). Finally, we sampled from this PDF to add jitter to the tissue photon packet ToF from the Monte Carlo simulation and generated apparent ToFs for each photon packet. The sampling of the random variables from the PDF has been done through the inverse transform sampling technique.^52^ Thus, for every detected photon packet, we have two ToFs: first, the tissue ToF, representing the ToF of the photon packet within the simulated tissue, and second, the apparent ToF that accounts for the extra time lag due to a non-ideal IRF. We use the apparent ToFs to allocate the detected photon packets to individual time gates, and then use the tissue path lengths of these allocated photon packets (along with their momentum transfer histories) to obtain the $G_{1}$ for the time gates using Eq. (1). To visualize the efficacy of photon selection, we evaluate the distribution of the detected photon fluence versus the photon path length in tissue. The time-dependent photon fluence was computed for every time gate to obtain the detected path length distribution function P(L) across the time gates (note this is different from the photon packet path length distribution as longer path lengths are attenuated more, thus shorter path lengths contribute disproportionately to the signal). The fluence Φ(t,t+Δt) accumulated within a time interval t to t+Δt of a time gate is computed with the following equation:^53^

$$\Phi (t,t+\Delta t)=\frac{1}{\Delta t}\overset{N(\Delta t)}{\underset{i=1}{\sum }}\overset{N_{\text{tissue types}}}{\underset{j=1}{\prod }}\exp(-\mu_{a,j}L_{i,j}),$$ (2)

where N(Δt) is the number of photon packets detected within the interval, $N_{\text{tissue types}}$ is the number of tissue types, and $\exp(-\mu_{a,j}L_{i,j})$ accounts for the effects of absorption in each of the tissue types with $L_{ij}$ being the path length of photon packet i through the tissue type j. P(ToF) is obtained by normalizing the time-dependent photon fluence Φ(t) by dividing it by the sum of the accumulated photon fluence in all the discrete time intervals. Finally, the path length distribution function P(L) was obtained by multiplying the photon propagation time by the speed of light in the medium.

Table 2 lists the names of the lasers and photodetectors that were used to obtain various experimentally derived IRFs using time-correlated single photon counting technique. The details of IRF measurement can be found in Tamborini et al.^30^ Apart from the experimental IRFs, we have considered Gaussian IRFs of various full-width-at-half-maximums (FWHMs) ranging from 200 to 450 ps. Based on the performance metrics across different time gates (shown in the Supplementary Material) for the Gaussian IRFs, we found the Gaussian IRF with FWHM of 300 ps offers the optimal balance between CBF sensitivity, SNR, and ability to reject influence of blood flow in the extracerebral layer across both wavelengths. Hence in this paper, we have considered a Gaussian shaped IRF with FWHM of 300 ps as the “ideal” IRF for *in-vivo* measurements of CBF using TD-DCS. To achieve a Gaussian IRF of FWHM of 300 ps with our lasers and detectors, we have shaped the laser pulse using an electro-optic modulator (EOM). Table 2 shows the list of all the IRFs that we have considered in this paper.

**Table 2:** The laser sources and detectors used for various IRFs at both the wavelengths.

Wavelength (nm)	Source	Detector	FWHM (ps)	Abbreviation
765	Picoquant VisIR-765-HP “STED” laser (VisIR)	MPD-fast gated detector (MPD-FG)	520	VisIR-FG
765	Picoquant’s VisIR-765-HP “STED” laser (VisIR)	MPD-red enhanced (MPD-RE)	530	VisIR-RE
765	Picoquant’s VisIR-765-HP “STED” laser (VisIR) with added EOM for shaping	MPD-fast gated detector (MPD-FG)	300	VisIR-FG with EOM
765	Picoquant’s VisIR-765-HP “STED” laser (VisIR) with added EOM for shaping	MPD-red enhanced (MPD-RE)	300	VisIR-RE with EOM
1064	PicoQuant’s CPDL-S-F/FA 1064-nm laser (CPDL 1064 laser)	Quantum Opus’ Opus One™ multi-channel near-infrared photon detector system, superconducting NW	510	1064-NW
1064	PicoQuant’s CPDL-S-F/FA 1064-nm laser (CPDL 1064 laser) with added EOM for shaping	Quantum Opus’ Opus One™ multi-channel near-infrared photon detector system, superconducting NW	300	1064-NW with EOM

#### 2.1.4 Defining time gates for TD-DCS simulations

**Fig. 2:**
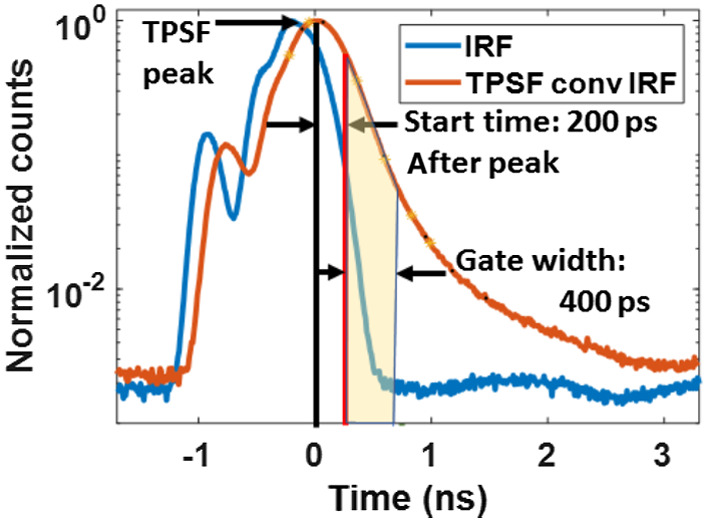
Photon allocation in time gates: showing IRF (blue) of an S–D pair with corresponding overall TPSF (orange) after convolution with IRF. The photons that fall in the transparent yellow area of the convolved TPSF are allocated to the time gate with start time of 200 ps and gate width of 400 ps. The ratio of this yellow area to the area under the convolved TPSF (orange) was used to determine the photon counts per second for the time gate, as mentioned in Sec. 2.1.6.

To identify the time gates for optimal performance of the TD-DCS technique, we have considered a wide range of feasible time gates. Each time gate is characterized by two independent parameters: gate start time and gate width. Photons were binned in time gates according to their apparent ToF. Once the photons are selected for each gate, their tissue path length and momentum transfer histories were used for the computation of $g_{1}$ (and β) for every time gate, and their path length weighted contributions were used to estimate the expected photon count for each time gate. Throughout our simulations, the start time of a gate was always defined with respect to the occurrence of the peak of the TPSF, consistent with the time gate definition used in our previous TD-DCS publication.^30^ A table of IRF to TPSF peak delays is provided in the Supplementary Material. Figure 2 illustrates the process of allocating detected photons into time gates.

#### 2.1.5 Computation of coherence factor (β)

The coherence factor (β) for each time gate was computed using the relationship for obtaining $g_{2}$ for a limited coherence light source proposed by Bellini et al.,^54^

$$g_{2}(\tau )=1+\int_{0}^{\infty }dL\int_{0}^{\infty }dL^{\prime }P(L)P(L^{\prime })g_{1}(L,\tau )g_{1}(L^{\prime },\tau )e^{-2{\left[(L-L^{\prime })/L_{c}\right]}^{2}},$$ (3)

where L represents the path lengths of the detected photons, P(L) is the path length distribution function, $g_{1}(L,\tau )$ is the path length-dependent field autocorrelation function, and $L_{c}$ is the coherence length of the incident light from the laser. To obtain β, we reduce Eq. (3) into the following expression:

$$\beta (\text{time gate})=g_{2}(\tau =0)-1=\int_{0}^{\infty }dL\int_{0}^{\infty }dL^{\prime }P_{\text{time gate}}(L)P_{\text{time gate}}(L^{\prime })e^{-2{\left[(L-L^{\prime })/L_{c}\right]}^{2}}.$$ (4)

The path length distribution P(L) was computed for each gate from the individual photon histories. P(L) can also explain the effect of IRF on BFi sensitivity to CBF and β. This will be discussed further in Sec. 3.2. We used a Michelson interferometer to determine the laser coherence length. Based on these measurements, we use a laser coherence length $L_{c}$ of 5 cm for simulations using an unshaped laser pulse IRFs and 4 cm for simulations with an EOM-shaped pulse IRFs. For Gaussian IRFs, the FWHM’s of the IRF and an assumed 90-ps FWHM Gaussian detector response are used to calculate the laser pulse width, which is then in turn used to directly compute $L_{c}$. For a transform-limited Gaussian pulse, the bandwidth of the pulse is given by BW (Δν)=0.441/FWHM. Finally, the $L_{c}$ for the Gaussian IRF was determined using the following relation: $L_{c}=c/\pi *\Delta \nu$, where c is the speed of light in tissue.

#### 2.1.6 Addition of noise

To simulate a realistic TD-DCS measurement, we added zero-mean Gaussian noise to the obtained $g_{2}$ based on a statistical noise model for correlation measurements^55^^,^^56^ giving the standard deviation of the noise [σ(τ)] at each time delay τ,

$$\sigma (\tau )=\sqrt{\frac{T}{t}}\;{\left[\beta^{2}\frac{(1+e^{-2\Gamma T})(1+e^{-2\Gamma \tau })+2m(1-e^{-2\Gamma T})e^{-2\Gamma \tau }}{\left(1-e^{-2\Gamma T}\right)}+2{\left\langle n\right\rangle }^{-1}\beta (1+e^{-2\Gamma \tau })+{\left\langle n\right\rangle }^{-2}(1+\beta e^{-\Gamma \tau })\right]}^{1/2},$$ (5)

where t is the averaging time, T is the bin width, Γ is the exponential decay rate of $g_{1}(\tau )$, ⟨n⟩ is the average number of photons within the bin width T (also given as ⟨n⟩=IT, where I is the detected photon count rate), m is the bin index, and β is the coherence factor.

The coherence factor, β, was obtained as described in Sec. 2.1.5. The detected photon counts I for each time gate have been obtained by taking the area under the TPSF curve within a given time gate. As per the ANSI standard limits for safe skin exposure (ANSI Z136.1), the maximum permissible exposure of skin by optical illumination at 765 and 1064 nm are 0.27 and $1.07\;W/{\mathrm{cm}}^{2}$, respectively. Following the standard, for an illumination spot larger than 1-mm diameter, a 3.5-mm aperture can be applied, translating the maximum permissible power to be 26 mW at 765 nm and 103 mW at 1064 nm. Based on these power limits, we typically inject 100 mW of laser power at 75-MHz laser repetition rate into the forehead of human subjects. At this input power, we are typically able to detect 4 million photons per second at 10-mm S–D separation. Similarly, for 765 nm, after inputting 26 mW of power to human head, we are typically able to collect 600,000 photons per second at 10-mm S–D separation with a distributed source arrangement where six source fibers were arranged in a 1-cm radius circle (at least 1 cm apart) around the detector location when using the MPD FG detector (this was done to compensate for the low photon detection efficiency of this detector). Therefore, we have used these count rates in our simulations. Specifically, we have assumed the total area under the TPSF to correspond to these photon counts.

### 2.2 Inverse Problem

To fit the $g_{2}(\tau )s$ for various time gates, obtained during the forward simulations, we modeled the light propagation in a homogeneous medium (as described in Secs. 2.1.3 and 2.1.4). The optical properties of the homogeneous medium, a semi-infinite slab in this case, were determined by fitting the obtained TPSF (from the forward model) to an equation describing the time-resolved solution of the diffusion equation. ^57^ Finally, the BFis and βs were obtained for each time gate after fitting the $g_{2}(\tau )s$ (obtained during the forward simulation) to the coupled equations: Eq. (1) and the Siegert relation $g_{2}(\tau )=1+\beta {\left|g_{1}(\tau )\right|}^{2}$. Note that the path lengths and the momentum transfers used in Eq. (1) during the fitting were obtained from Monte Carlo simulation of photon propagation in the homogeneous medium.

The non-linear optimization was accomplished using the “lsqnonlin” subroutine in Matlab^®^ that is based on the Levenberg Marquadt algorithm. While fitting for both the noisy and noiseless $g_{2}s$, a set of two initial guesses each for BFi and β (four combinations) have been used. Finally, out of the recovered BFi and β values, the pair with least Euclidean norm of the residuals was chosen.

### 2.3 Computation of Intrinsic Sensitivity

To distinguish the blood flow in the brain and in the extracerebral layers, we have considered the hemodynamic processes in the skin (Sn), skull (Sl), and cerebral spinal fluid (CSF) as the extra-CBF and the blood flow in the gray matter and white matter as brain blood flow or CBF. Next, we simulated two different hemodynamic conditions to determine the sensitivities of the recovered BFi to CBF at various time gates. The first is the baseline condition, where the effective diffusion coefficient D is maintained at $1\times 10^{-6}\;{\mathrm{mm}}^{2}/s$ in the Sn and $6\times 10^{-6}\;{\mathrm{mm}}^{2}/s$ in the brain. The second is the perturbed condition, where the D value in the brain has been incremented by 20% to $7.2\times 10^{-6}\;{\mathrm{mm}}^{2}/s$, but the D value for the Sn layer remains unchanged. We assumed zero dynamics in the Sl^26^^,^^58^ and CSF layer. Using these dynamics within the segmented head, we computed the $g_{2}$ for various time gates for both the baseline and the perturbed conditions, as explained in Sec. 2.1.

Once the $g_{2}s$ are obtained for various time gates for the baseline and perturbed conditions, they are fitted to recover BFi and β. Finally, the intrinsic sensitivity is computed using Eq. (6):

$$\text{Intrinsic sensitivity of a time gate}\;(\%)=\frac{({\mathrm{BFi}}_{\text{perturbed}\_\text{brain}}/{\mathrm{BFi}}_{\text{baseline}})-1}{({\mathrm{CBF}}_{\text{perturbed}}/{\mathrm{CBF}}_{\text{baseline}})-1}\times 100,$$ (6)

where ${\mathrm{BFi}}_{\text{perturbed}\_\text{brain}}$ and ${\mathrm{BFi}}_{\text{baseline}}$ are the recovered BFis of the time gate during the perturbed and baseline conditions, respectively; ${\mathrm{CBF}}_{\text{perturbed}}$ and ${\mathrm{CBF}}_{\text{baseline}}$ are the D values in the brain during the perturbed and baseline conditions, respectively.

Another important parameter for evaluating the efficacy of a time gate is the ability to reject the influence in measured BFi due to hemodynamic processes in the extra-cerebral layers. To this end, we had adopted a similar approach as intrinsic sensitivity computation. Here, we have the same pair of D values for the brain and Sn layer during the baseline condition as above; however, for the perturbed condition, we have incremented the blood flow of Sn layer by 20% to $1.2\times 10^{-6}\;{\mathrm{mm}}^{2}/s$, keeping the D value in the brain the same as baseline. With these dynamics, we compute the $g_{2}s$ across various time gates for both the baseline and perturbed conditions as before. Then we fit the obtained $g_{2}s$ for the set of BFis for both the conditions and compute superficial sensitivity using the following equation:

$$\text{Superficial sensitivity of a time gate}=\frac{({\mathrm{BFi}}_{\text{perturbed}\_\text{superficial}}/{\mathrm{BFi}}_{\text{baseline}})-1}{(\text{superficial}\_D_{\text{perturbed}}/\text{superficial}\_D_{\text{baseline}})-1}\times 100,$$ (7)

where ${\mathrm{BFi}}_{\text{perturbed}\_\text{superficial}}$ and ${\mathrm{BFi}}_{\text{baseline}}$ are the BFis that are recovered during perturbed and baseline conditions, respectively; $\text{superficial}\_D_{\text{perturbed}}$ and $\text{superficial}\_D_{\text{baseline}}$ are the D values of the Sn layer during the perturbed and baseline conditions, respectively, while evaluating superficial sensitivity. The recovery of BFi and β for the time gates has been discussed in Sec. 2.2. Both the intrinsic and superficial sensitivity were obtained with noiseless $g_{2}$.

### 2.4 Computation of Contrast-to-Noise Ratio and Figure-of-Merit

The intensity autocorrelation functions, $g_{2}$, computed for every time gate had random noise added based on the noise model described in Sec. 2.1.6. We have generated a total of 120 $g_{2}s$, each with an integration time of 1 s, and added a corresponding noise realization to each to simulate a 120-s long measurement. These noisy $g_{2}s$ were then fitted to retrieve a time series of BFis and βs as explained in Sec. 2.2. To determine the contrast-to-noise ratio (CNR) for a time gate, we divide the change in recovered BFi from the 20% change in brain blood flow by the standard deviation of the recovered BFis over the 120 time points [Eq. (8)]. The 20% perturbation was chosen to reflect a physiologically significant blood flow change. In our simulations, we have accounted for physiological noise by setting the coefficient of variation (CoV, the ratio of standard deviation to the mean) of the BFi time series to be at least 0.1 at all gates, as observed by our group during measurements on human subjects.

$$\mathrm{CNR}(\;\text{time gate})=\frac{{\mathrm{BFi}}_{\text{perturbed}\_\text{brain}}-{\mathrm{BFi}}_{\text{baseline}}}{\text{standard deviation}\;\text{of}\;120\;\text{recovered}\;\mathrm{BFi}}.$$ (8)

To take into account the impact of extra-cerebral layer cross-talk, we further defined a figure-of-merit (FoM) that takes into account a time gate’s CNR [from Eq. (8)] as well as its ability to reject the influence of changes in the blood flow of the extra-cerebral layer [from Eq. (7)]:

$$\mathrm{FoM}\;(\text{time gate})=\frac{\mathrm{CNR}\;(\text{time gate})}{\text{Superficial sensitivity}\;(\text{time gate})/100}.$$ (9)

### 2.5 Experimental Verification of Predicted Noise Levels

**Fig. 3:**
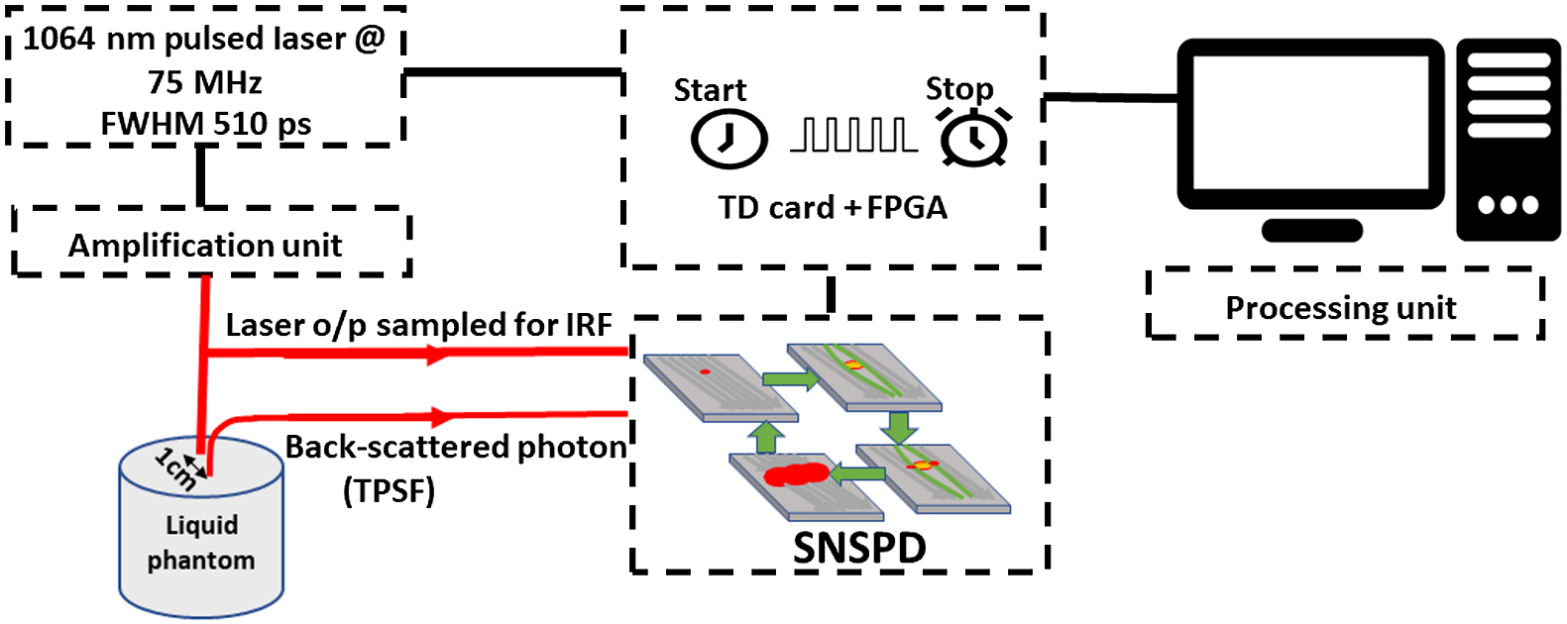
The optical TD configuration. The amplified seed laser illuminated the phantom, one NW channel collects the TPSF and the second one collects the IRF. The readout data are transferred to the TDC cards and are analyzed via a processing unit.

To experimentally validate the presented Monte Carlo analysis, we used a TD-DCS system operating at 1064 nm. The system consisted of a PicoQuant pulsed laser (CPDL 1064 laser, ∼510  ps FWHM) at repetition rate of 75 MHz that was amplified using a Yb-doped fiber amplifier and used to illuminate an intralipid-based phantom ($\mu_{a}=0.14\;{\mathrm{cm}}^{-1}$, ${\mu_{s}}^{\prime }=4.1\;{\mathrm{cm}}^{-1}$) via multi-mode fiber (62.5/125  μm). The optical power was set to collect 3M counts per second at 10-mm S–D distance. The backscattered photons were collected via single-mode fibers and coupled into the nanowire (NW) detector (Quantum Opus, Opus One) with a photon detection efficiency >80%, dark count <100  counts/s, and jitter <100  ps. The photons were collected via two channels, one for the IRF and one for the TPSF. The NW readout electronics module is connected to a custom-made set of time-to-digital converter cards (10-ps resolution, 40-ps FWHM jitter) and to a 150-MHz clock rate time-tagger as previously reported for our TD-DCS implementation.^30^ The schematic of our experimental setup is shown in Fig. 3. This corresponds to the 1064-NW entry in Table 2.

## 3 Results

### 3.1 IRFs and the Corresponding TPSFs

**Fig. 4:**
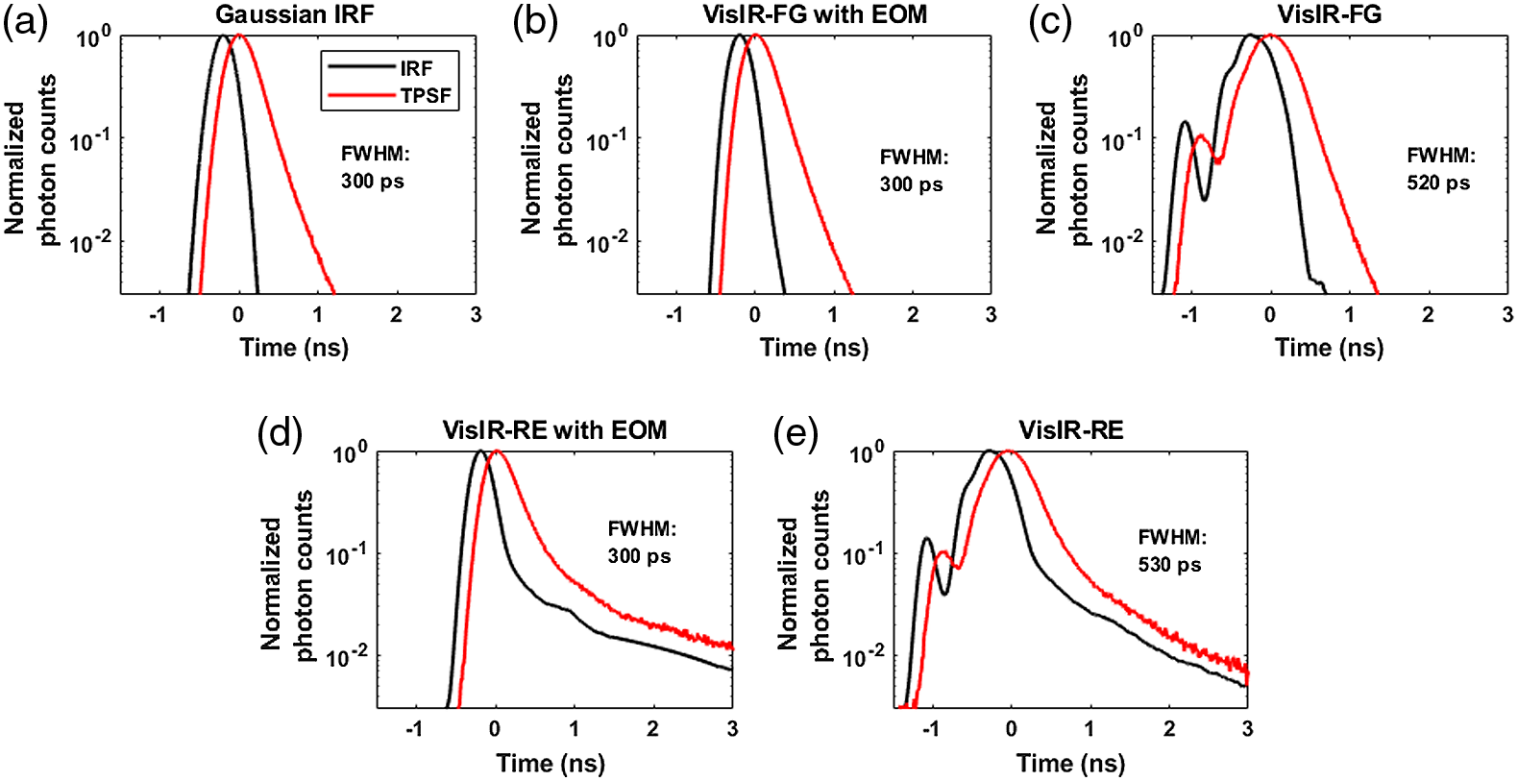
Showing all the IRFs [all measured except (a)] at 765 nm and the corresponding TPSFs: (a) Gaussian IRF, (b) VisIR-FG with EOM, (c) VisIR-FG, (d) VisIR-RE with EOM, and (e) VisIR-RE.

**Fig. 5:**
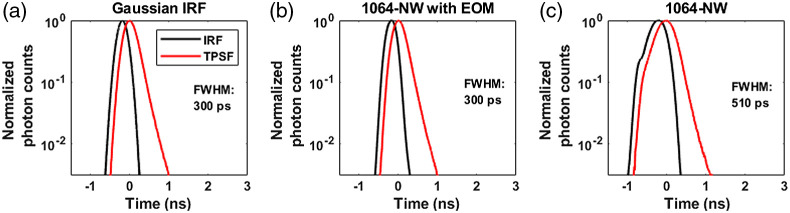
IRFs [all measured except (a)] at 1064-nm excitation wavelengths: (a) Gaussian IRF, (b) 1064-NW with EOM, and (c) 1064-NW.

Figures 4 and 5 show the measured and synthetic IRFs for 765- and 1064-nm simulations, respectively, along with the corresponding convolved overall-TPSFs. The IRFs that were obtained from combinations of commercially available sources and detectors are shown in Figs. 4(b)–4(e), 5(b), and 5(c). The RE detector was considered here because it has substantially higher (6 to 7×) photon detection efficiency at 765 nm compared the FG detector. However, as can be seen in Figs. 4(d) and 4(e), the RE detector exhibits a long diffusion tail, substantially broadening the IRF. As explained in Sec. 2, to achieve narrower IRFs for attaining better sensitivity to CBF, we also considered the use of an EOM to shape the laser pulse as shown in Figs. 4(b), 4(d), and 5(b).

### 3.2 Time of Flight Distribution within Tissue

**Fig. 6:**
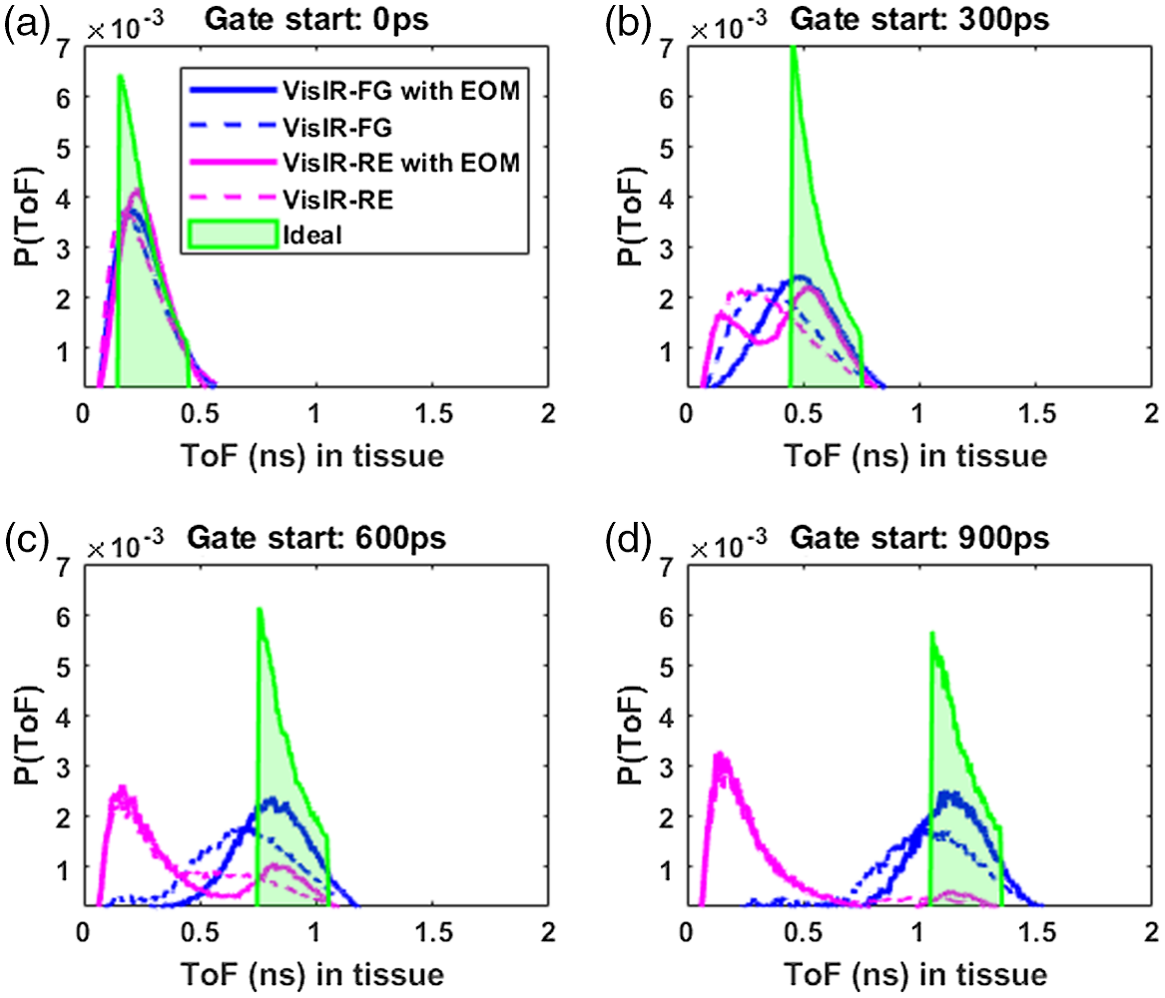
ToF distribution within tissue at different time gates with gate width of 300 ps for experimental IRFs at 765 nm and the “perfect” δ function IRF. Legend: solid blue for VisIR-FG with EOM, dotted blue for VisIR-FG, solid magenta for VisIR-RE with EOM, dotted magenta for VisIR-RE, and green shade for the δ function IRF.

The tissue photon time of flight distribution functions, P(ToF), was obtained as described in Sec. 2.1.3 for all time gates. P(ToF) of a time gate gives the actual tissue ToF probability distribution for photons allocated to that time gate based on the overall TPSF. P(ToF) does not match the desired gating profile due to non-idealities in the system IRF. To understand the effect of different IRFs on the apparent ToF distribution, we use the experimentally derived IRFs and a “perfect” δ-function IRF at 765 nm to compare the actual tissue ToF at several time gates selected from the overall TPSF. Figure 6 shows the comparison of P(ToF)s for the IRFs across four different time gates with a fixed gate width of 300 ps. It can be seen from Fig. 6 that in the later time gates, i.e., the time gates with start times of 600 and 900 ps, there is “leak-through” of photons with shorter travel time within the tissue because of the IRF profile. For the VisIR-RE IRF, the number of early photons in fact ends up exceeding the number of photons that have a tissue ToF falling within the desired time gate. Figure 6 also illustrates that shaping the laser pulse to obtain a near Gaussian-shaped IRF with a narrow width and using a photodetector that does not add a long diffusion tail to the IRF is needed to be able to effectively select photons with longer ToFs in tissue at later gates.

### 3.3 Coherence Factor (β)

**Fig. 7:**
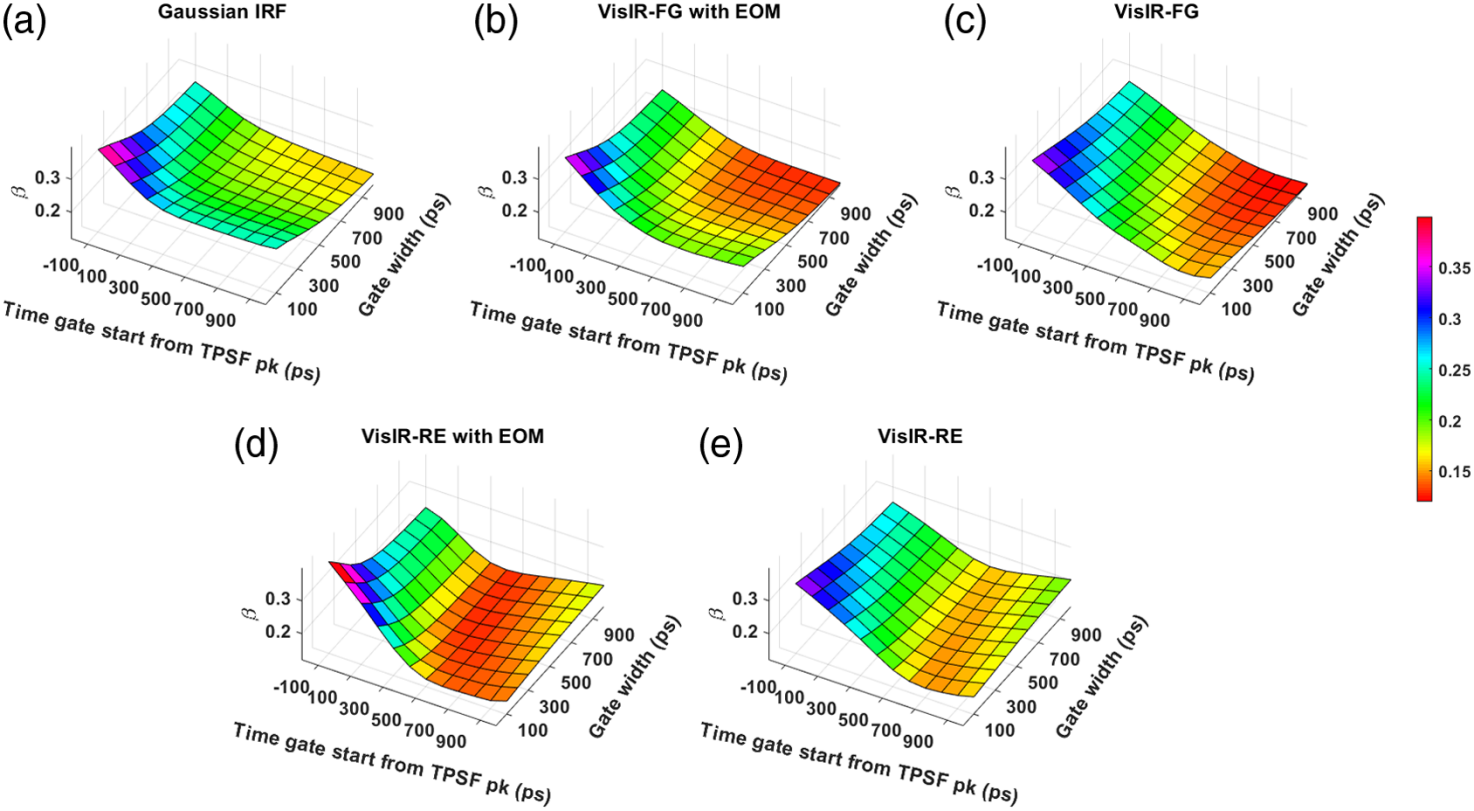
Variation of β across times gates for different IRFs at 765 nm: (a) Gaussian IRF, (b) VisIR-FG with EOM, (c) VisIR-FG, (d) VisIR-RE with EOM, and (e) VisIR-RE.

**Fig. 8:**
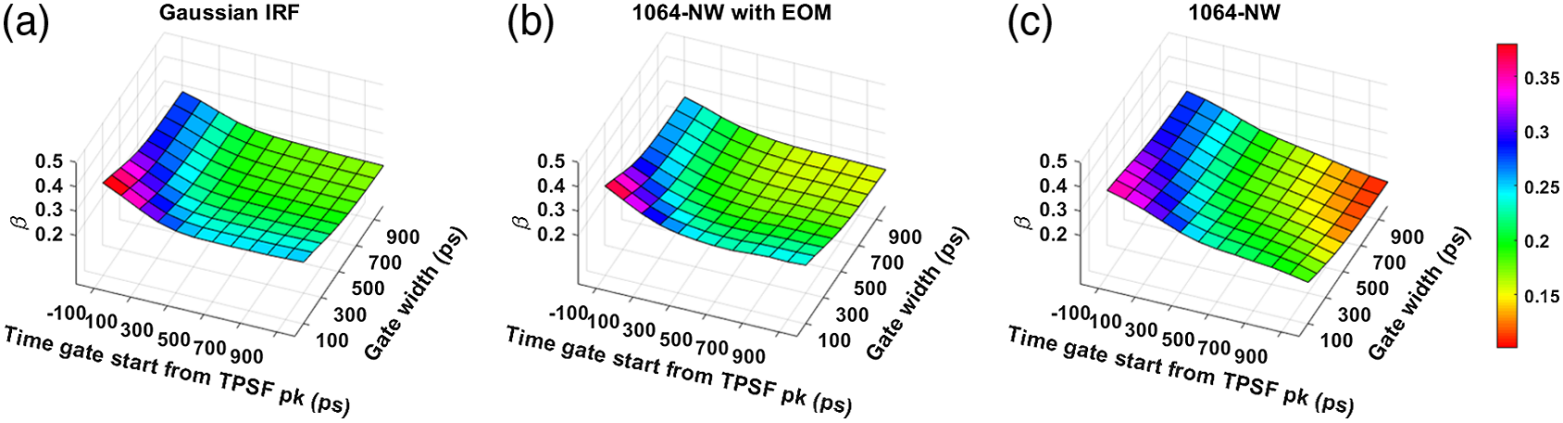
Variation of β across times gates for different IRFs at 1064 nm: (a) Gaussian IRF, (b) 1064-NW with EOM, and (c) 1064-NW.

Figures 7 and 8 show the computed coherence factor, β, across various time gates for different IRFs at 765- and 1064-nm excitation wavelengths, respectively. We observe that for every gate start time, β decreases with increasing gate width for all IRFs. The use of MPD-RE as detector leads to attaining lower values of β. This decrease starts at earlier time gates compared to when the MPD-FG was used due to the mixing of less coherent photons with an excessively different ToF which resulted from the poor ToF gating effectiveness shown in the previous section.

### 3.4 Intrinsic Sensitivity

**Fig. 9:**
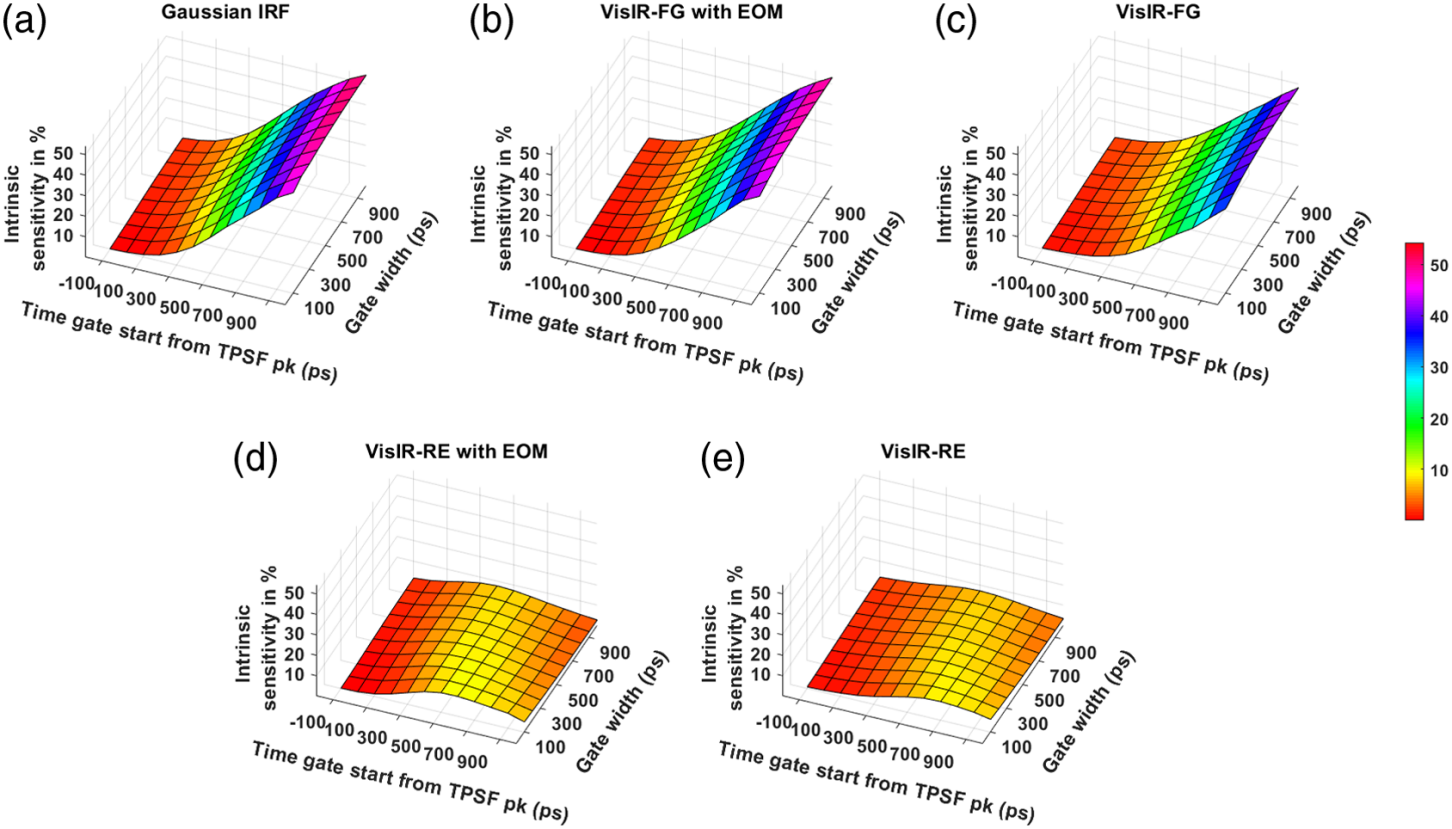
Intrinsic sensitivities across time gates for different IRFs at 765 nm: (a) Gaussian IRF, (b) VisIR-FG with EOM, (c) VisIR-FG, (d) VisIR-RE with EOM, and (e) VisIR-RE.

**Fig. 10:**
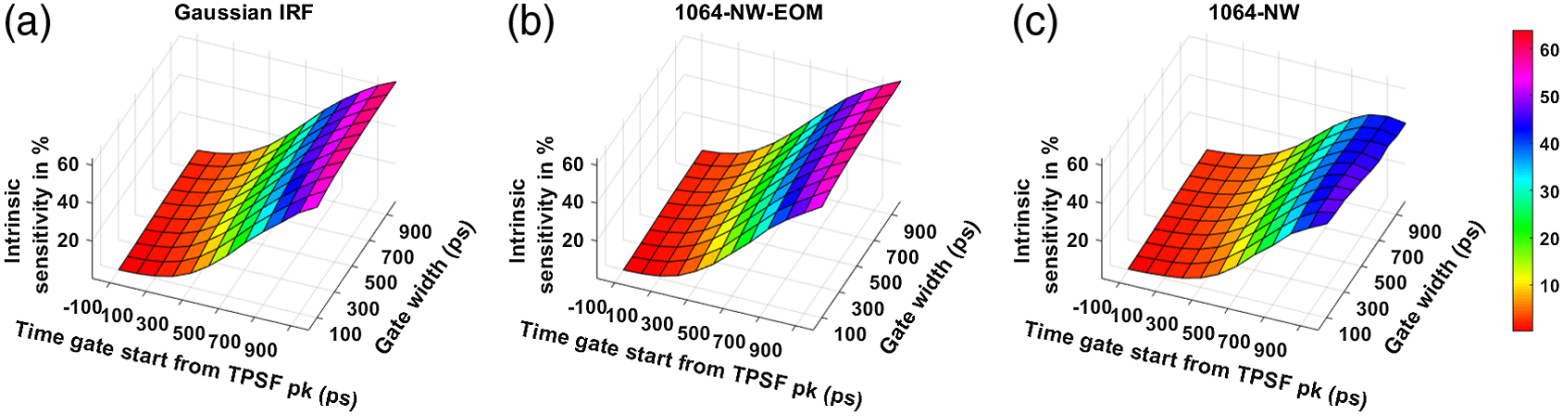
Intrinsic sensitivities across time gates for different IRFs at 1064 nm: (a) Gaussian IRF, (b) 1064-NW with EOM, and (c) 1064-NW.

The noise-less intrinsic sensitivity across the time gates for different IRFs at the two wavelengths is shown in Figs. 9 and 10. Figures 9(d) and 9(e) show that using the MPD-RE detector, we cannot achieve a BFi sensitivity to CBF that is more than 9% to 10%, for any of the time gates due to the short path photon leak-through. Comparing Figs. 9(b) and 9(c), we can see that using an EOM to shape the laser pulse helps in improving the sensitivity to CBF with the later time gates. Figure 10 shows that a further improvement of sensitivity can be achieved with excitation wavelength of 1064 nm due to higher penetration into the brain because of lower effective attenuation coefficient compared to 765 nm (Table 1). For instance, at 1064-nm excitation wavelength, a Gaussian IRF of 300-ps FWHM (and 1064-NW with EOM IRF) can make it possible to achieve a sensitivity as high as around 64% [Fig. 10(a)] with one of the later time gates. On the other hand, with 765-nm excitation wavelength, a maximum sensitivity of around 54% can only be attained with a Gaussian IRF of 300 ps FWHM (and VisIR-FG with EOM IRF).

### 3.5 Contrast-to-Noise Ratio

**Fig. 11:**
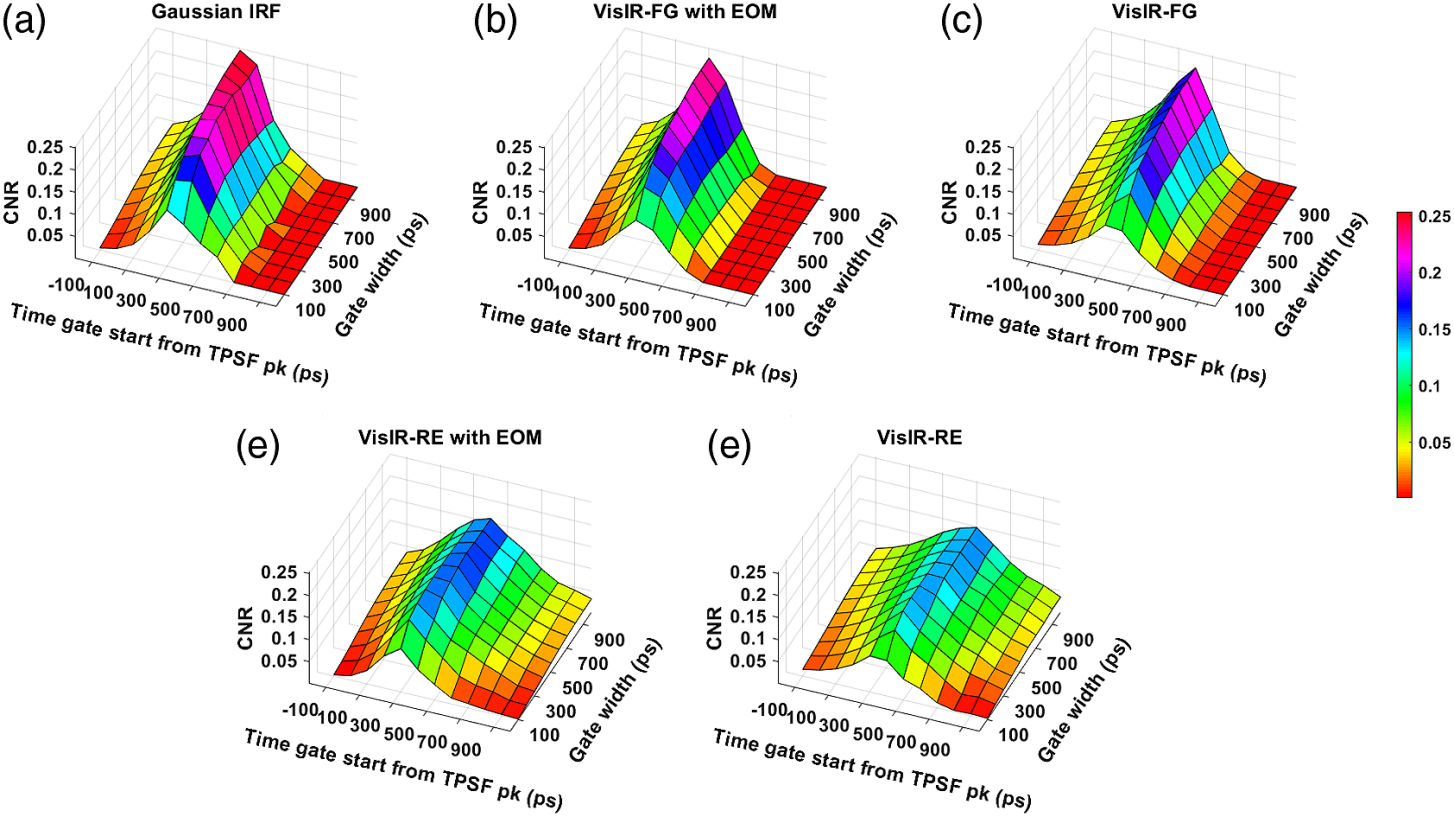
CNR across time gates for different IRFs at 765 nm: (a) Gaussian IRF, (b) VisIR-FG with EOM, (c) VisIR-FG, (d) VisIR-RE with EOM, and (e) VisIR-RE.

**Fig. 12:**
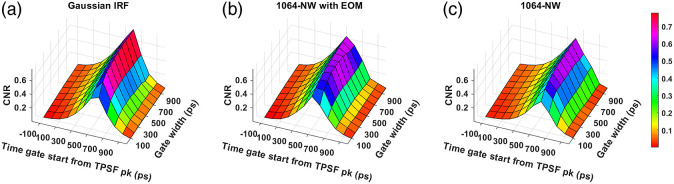
CNR across time gates for different IRFs at 1064 nm: (a) Gaussian IRF, (b) 1064-NW with EOM, and (c) 1064-NW.

The obtained CNR across time gates for various IRFs have been shown in Figs. 11 and 12 for excitation wavelengths of 765 and 1064 nm, respectively. For all the IRFs at 765 nm, we observe that the CNR decreases at the late time gates, unlike the intrinsic sensitivity. We find that at 765 nm, the experimental IRF with the highest CNRs is VisIR-FG with EOM. For this IRF, we find that the upper 20% of CNR values are found for time gates starting at 300 ps with gate widths of 500 ps and above. For 1064 nm, for the best performing experimentally measured IRF, i.e., 1064-NW with EOM, we find that the region of time gates with highest CNR is now at gate start times from 500 to 600 ps and with gate widths of 300 ps and above. For the synthetic Gaussian IRF at 1064 nm, the optimal gate start time occurs approximately 100 ps later.

### 3.6 Sensitivity to Change in Extracerebral Hemodynamics

**Fig. 13:**
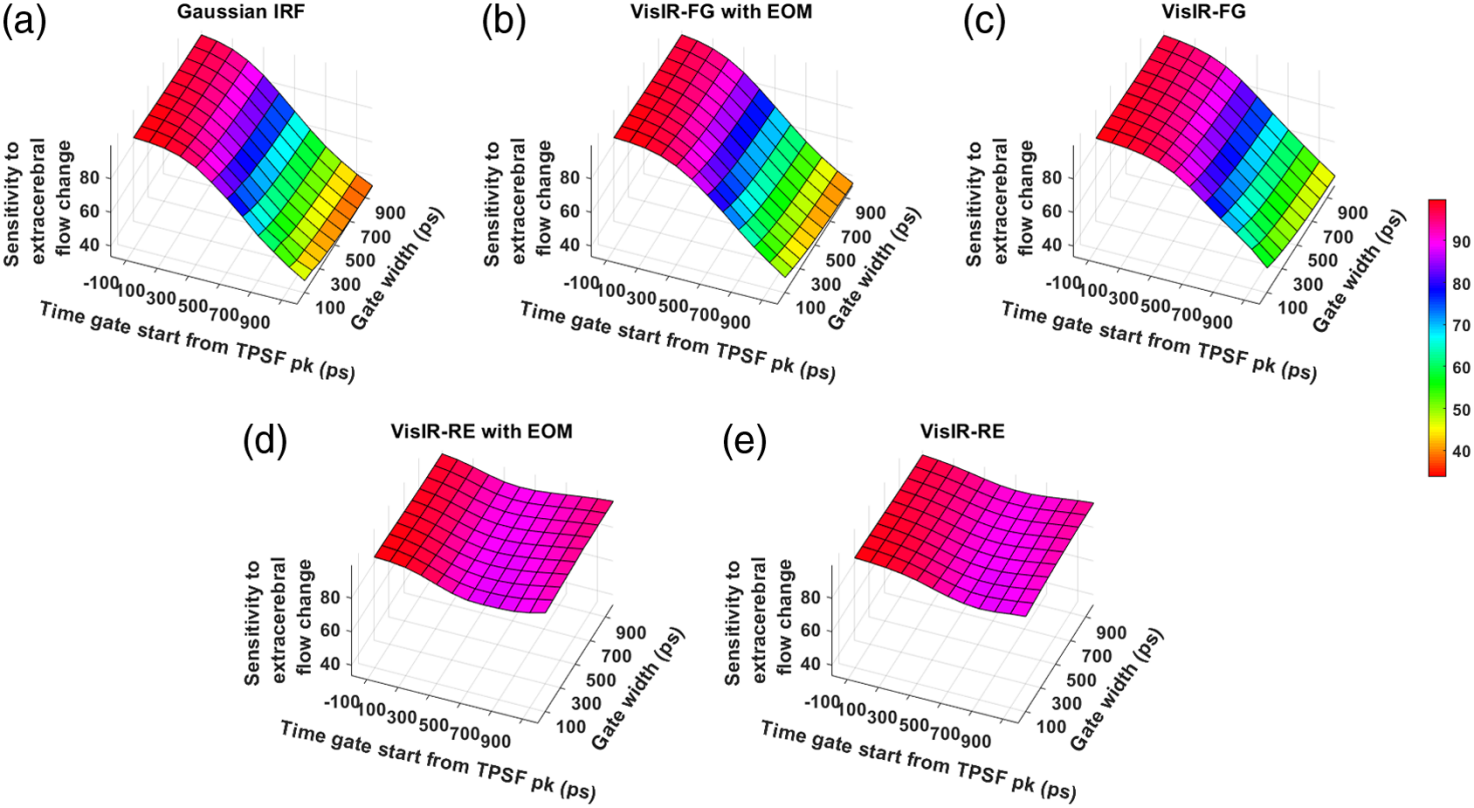
Sensitivity to change in extracerebral across time gates for different IRFs at 765 nm: (a) Gaussian IRF, (b) VisIR-FG with EOM, (c) VisIR-FG, (d) VisIR-RE with EOM, and (e) VisIR-RE.

**Fig. 14:**
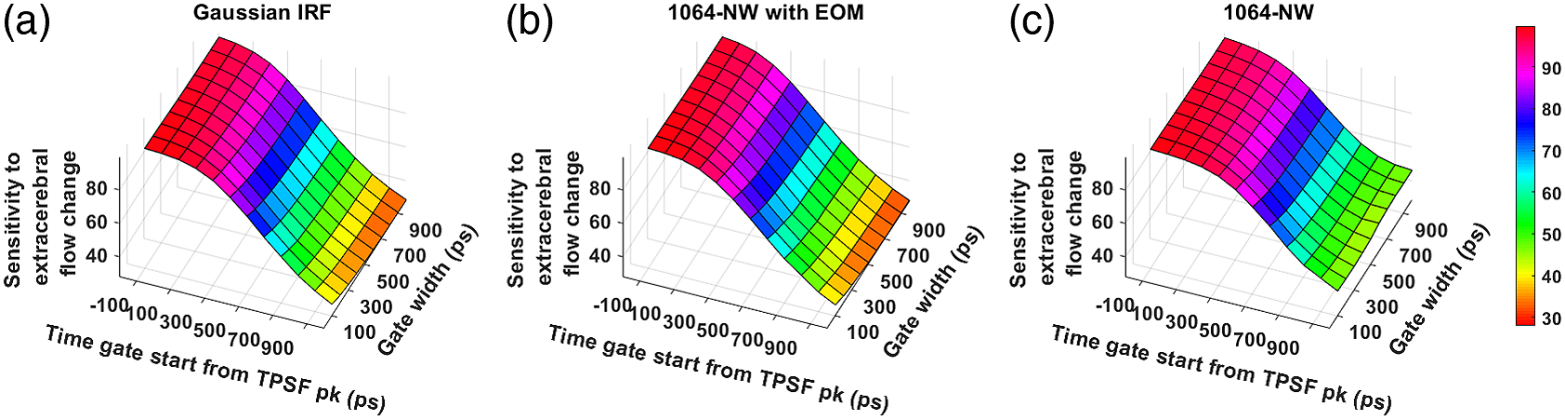
Sensitivity to change in extracerebral across time gates for different IRFs at 1064 nm: (a) Gaussian IRF, (b) 1064-NW with EOM, and (c) 1064-NW.

The sensitivity of BFi to change of blood flow in the extracerebral layer for different IRFs across various time gates at 765 and 1064 nm are shown in Figs. 13 and 14, respectively. Here, we find that the later gates are useful in reducing the influence of extra-CBF variation for all the IRFs, except the VisIR-RE and the VisIR-RE with EOM. While comparing the sensitivities of the Gaussian IRFs across wavelengths [Figs. 13(a) and 14(a)], we observe a slight improvement at 1064 nm in terms of being less sensitive to a perturbation in extra-CBF at the later time gates.

### 3.7 Figure of Merit

**Fig. 15:**
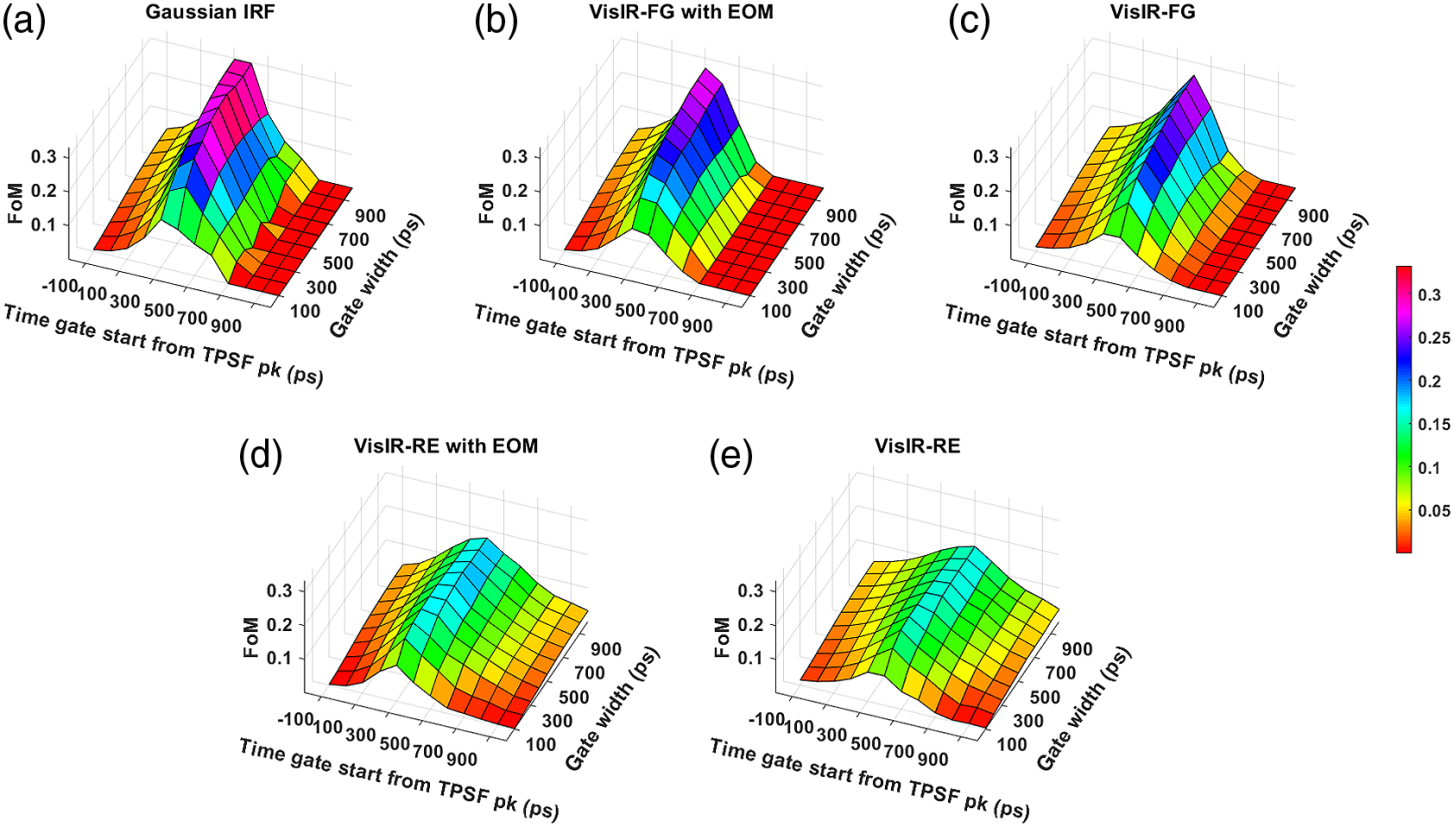
FoM across time gates for different IRFs at 765 nm: (a) Gaussian IRF, (b) VisIR-FG with EOM, (c) VisIR-FG, (d) VisIR-RE with EOM, and (e) VisIR-RE.

**Fig. 16:**
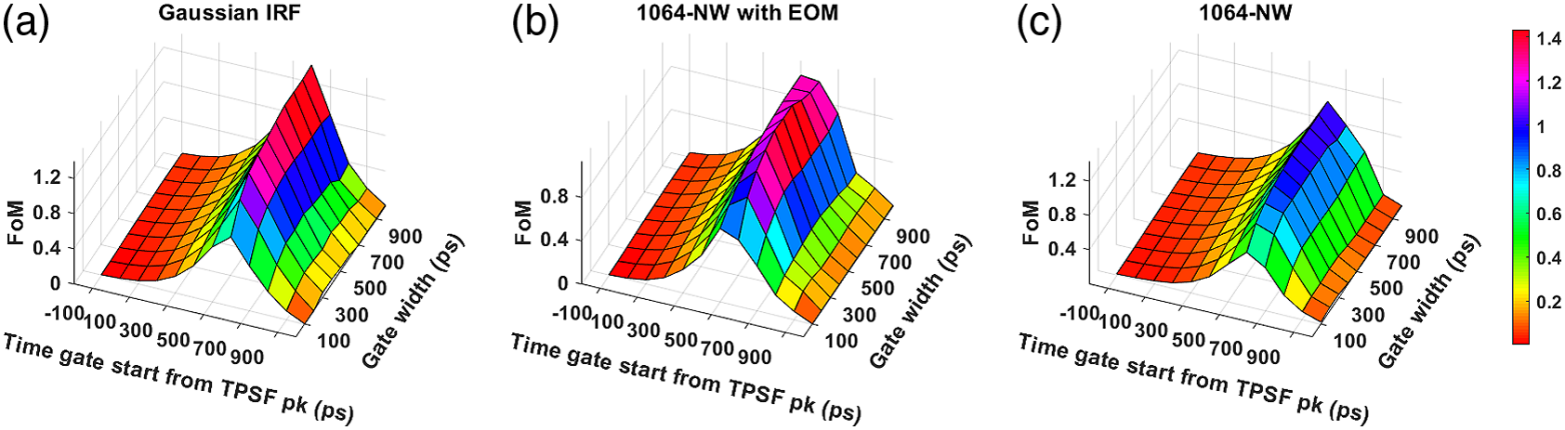
FoM across time gates for different IRFs at 1064 nm: (a) Gaussian IRF, (b) 1064-NW with EOM, and (c) 1064-NW.

Figures 15 and 16 show our final metric for comparing the performance across various time gates, the FoM that combines the influence of intrinsic sensitivity, noise, and extracerebral crosstalk potential for 765 and 1064 nm, respectively. We find that the best-performing hardware-based IRF at 765 nm corresponds to the VisIR-FG with EOM configuration based on the FoM metric. We find that the upper 20% of FoM values occur for time gates with gate start times between 300 and 400 ps after the TPSF peak and with a width of 500 ps and larger at 765 nm. For 1064 nm, for the best-performing hardware-based IRF, 1064-NW with EOM, the time gates start from 500 to 600 ps with gate width of 500 ps and larger.

### 3.8 Comparison with Thicker Extracerebral Layer

**Fig. 17:**
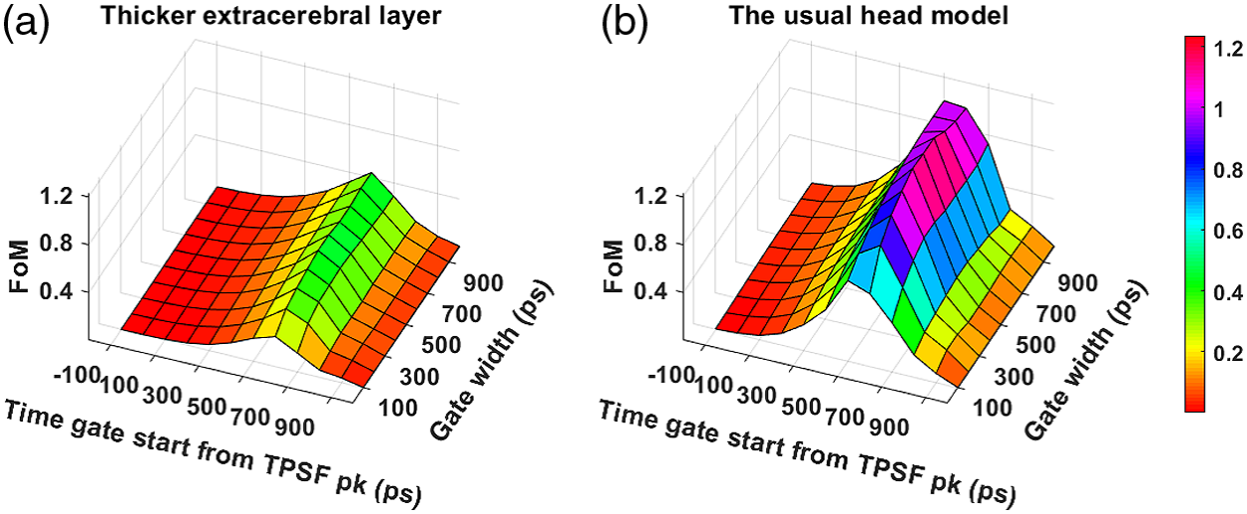
Comparison of FoM across various time gates for 1064-NW with EOM IRF at 1064 nm on human head model with (a) thicker extracerebral layer and (b) usual extracerebral layer.

To study the effect of extracerebral thickness on the optimal range of time gates, we have repeated our analysis on a human head geometry with extracerebral thickness increased by 3 mm. Figure 17 shows the comparison of the obtained FoM across the time gates at 1064 nm wavelength with the best-performing experiment-based IRF, 1064-NW with EOM. We see that the distribution of FoM values across the gate start and width matrix is very similar between the two scenarios. As expected, we notice the absolute performance values being significantly reduced for the thicker extracerebral layer scenario.

### 3.9 Experimental Verification of β and the Noise Level

**Fig. 18:**
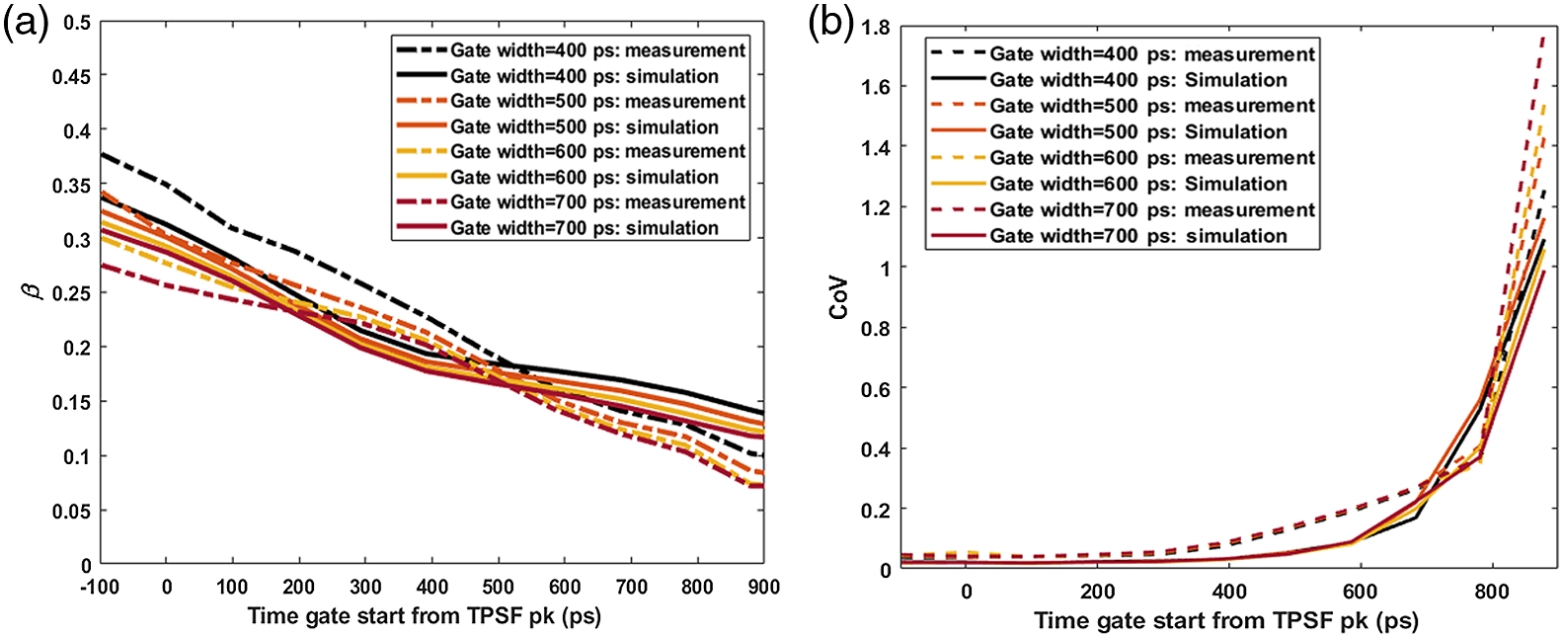
Comparison of (a) β and (b) CoV across various time gates from the measurements (dotted lines) and the corresponding gates from the simulations (solid lines).

To verify the simulated TD-DCS operating conditions are realistic, we compared β and the CoV, defined as the ratio of the standard deviation to the mean, of the recovered ${\mathrm{BF}}_{i}$ time-series between simulations and liquid phantom experiments (here, reflecting Brownian diffusion) across the time gate matrix. For these simulations, we used a semi-infinite geometry with the same optical properties as the phantom and matching photon counts as seen in the experiment. Figure 18(a) shows the comparison of the obtained β from simulations and liquid phantom measurements. In Fig. 18(b), we compare the obtained BFi CoVs from simulations and liquid phantom measurements.

## 4 Discussions

From the simulations, we found that the overall TD-DCS performance is heavily dependent on the characteristics of the laser source and photodetector. Hence, we began by comparing the performance across the time gates for a number of Gaussian shaped IRFs of varying width (shown in the Supplementary Material). From this exploration, we learned that ∼300  ps FWHM Gaussian IRF provides the best performance from the point of view of achieving the highest FoM values. We used this as a realistic reference (as opposed to a δ-function IRF used by previous work^31^) for comparing the performance and optimal time gates for hardware-driven IRFs and to provide a target for optimizing actual instrument IRFs using electro-optical laser shaping. The determination of the “ideal” IRF is important for choosing the proper source and detector combination for TD-DCS measurements. For instance, as also mentioned in our previous publication,^30^ while the custom silicon, enhanced red sensitivity MPD-RE detector has a quantum efficiency over 40% at 765 nm, the slow impulse response decay due to charge diffusion delays in the substrate prevents the efficient long path photon selection. Moreover, as seen in Figs. 9(d) and 9(e) and due to the broad system IRF achieved when using this detector, none of the time gates yield more than 10% sensitivity to brain blood flow, superficial cross-talk remains substantial even at late gates [see Figs. 13(d) and 13(e)], and the mixing of early and late photons leads to decreased coherence [manifested as low β, see Figs. 7(d) and 7(e)], significantly reducing achievable SNR. On the other hand, the use of a detector with sharp temporal response, such as the MPD Fast Gated detector, leads to significantly improved performance in photon selection. However, the limited quantum efficiency (as low as 5% to 7% at 765 nm) requires the use of a distributed source to ensure enough photons are detected while remaining under the regulatory exposure limits.

Another experimental parameter with significant impact on the optimal gating strategy is the temporal shape of the pulsed laser source. TD-DCS requires a delicate balancing act—using a pulse long enough to have several cm of coherence length, yet short enough to enable P(ToF) selectivity. The few hundred ps regime that meets these requirements is an area sparsely covered by commercial sources, if at all. The Picoquant VisIR-STED that our group used in implementing TD-DCS comes quite close, though the native pulse is longer than optimal. By shortening the pulse using an EOM [as displayed in Figs. 4(b) and 4(d)], we have shown that we can noticeably improve the intrinsic sensitivity at the later gates. Comparing the P(ToF)s in Fig. 6 between IRFs: VisIR-FG (in dotted blue) and VisIR-FG with EOM (in solid blue), we find that shaping the laser pulse helps in rejecting the early photons at the late gates [Figs. 6(c) and 6(d)]. Not only does this help in having better sensitivity to CBF at the later time gates but also this may improve β by minimizing the probability of mixing early photons with the later ones as seen in Fig. 6. It is also important to note that shortening of the laser pulse also leads to the reduction of laser coherence length—the pulse length needs to remain long enough to avoid losses due to reduced coherence length overcoming the benefits from better photon ToF selectivity.

We find that with almost all the IRFs (except those involving the MPD-RE detector), the intrinsic sensitivities (Figs. 9 and 10) increase monotonically with increasing gate start times. However, this metric cannot be solely used for determining the optimal time gates for measuring CBF using TD-DCS because it does not consider the effects of noise. When the CNR is considered instead, the late time gates do not offer the best performance (Fig. 11)—while the intrinsic sensitivity increases approximately linearly with gate start time, the noise impact grows rapidly. Figure 11 also shows that wider time gates are also beneficial—this is likely because the increase photon count leads to a reduction in noise that is more powerful than any associated decrease in β. For the best-performing IRF at 765 nm, the 300-ps FWHM VisIR-FG with EOM, the time gates with gate start times varying from 300 to 400 ps and widths of 500 ps and above correspond to the both the highest CNR and FoM. At 1064 nm, where a higher photon count is available, this range shifts to later time gates, having gate start times ranging from 500 to 600 ps and gate widths of 500 ps and higher for the best-performing measured IRF, 1064-NW with EOM, at that wavelength [Figs. 12(b) and 16(b)]. This shift of the high CNR/FoM region to the later time gates at 1064 nm indicates 1064-nm measurements can effectively exploit higher CBF sensitivity. Moreover, the results at 1064 nm show a 4 to 5 times improvement in FoM versus 765 nm when the 300-ps FWHM 1064-NW with EOM IRF is used. The β and the CNR/FoM of the Gaussian IRF look slightly better than the shaped IRF of the corresponding wavelength because the laser coherence length was around 4.6 cm for the former and 4 cm for the latter.

The optimization of gate settings for TD-DCS has been previously reported using a simplified approach that did not consider system IRF or statistical correlation noise models. Qiu et al.^31^ reports optimal gate starts of 700 to 800 ps with respect to the start of the TPSF—corresponding to gate starts of 560 to 660 ps with respect to the TPSF peak—and a gate width of 800 ps at 850 nm using a δ-function IRF (which is not achievable in practice). These gate opening times are quite different from our results (optimal gate start delays of 300 to 400 ps) at 765-nm wavelength in case of the best practically achievable IRF (VisIR-FG with EOM). The most likely reason for the disagreement of our gate opening times with the ones in Qiu et al.^31^ is that we have considered a real IRF as opposed an ideal one. Moreover, in our work, we have estimated the systemic noise to the measurements using a stochastic noise model, and we have also considered the effect of finite coherence length. Another reason for the difference in results is that we used a 1-s integration time in our simulations as opposed to an integration time of 5 s that was used in Qiu et al.^31^ A higher integration time will shift the optimal CNR to somewhat later gates but would be less suited for following fast CBF events.

The overall absolute FoM values across time gates dropped for the larger extracerebral thickness, as shown in Fig. 17(a). However, we notice that the optimal time gate for the head model with thicker extracerebral layer occurs at the same time as the baseline scenario, which implies that the optimal time gate can be largely chosen regardless of the subject characteristics. We also verified that the optimal time gates do not change if the probe is moved to another position (∼3  cm away) on the forehead. However, as expected, the absolute magnitude of the performance metrics does change depending on the thickness of the extracerebral later in the probed region on the forehead.

To ensure our simulations are reflective of experimental realities, we have compared βs and BFi CoVs, obtained from a measurement on a liquid phantom with those recovered from simulations with parameters matching the measurement conditions. As shown in Fig. 18(a), we found that the βs across different time gates obtained from the simulations are a reasonably good match for those obtained from measurements. We observed that the βs from the measurement decay little faster than the computed ones as we move the gate opening times away than from the peak of the TPSF. In both the cases, they reach a steady state value. Figure 18(b) shows that the CoVs across different time gates obtained from the simulations are a good match for those obtained from measurements.

## 5 Conclusion

We used Monte Carlo simulations on a realistic human head geometry to determine the time gate(s) for optimal performance of TD-DCS with commercially available laser sources and photon counting detectors at 765- and 1064-nm wavelengths. For the first time, we have quantified the IRF (driven by source and detector characteristics) influence on the performance of TD-DCS measurements using simulations representative of experimental conditions. We further show that electro-optical IRF shaping can lead to results close to an ideal Gaussian IRF. Once the IRF has been optimized, the choice of time gates balances increasing sensitivity with increasing noise at later gate starts, allowing a preferred gating strategy to be defined. Somewhat counterintuitively, wider gates are beneficial due to their positive impact on detected photon counts despite lower coherence. The optimal time gate(s) were determined for both wavelengths based on the key factors that affect the effectiveness of CBF monitoring using TD-DCS. The close agreement between simulation parameters and those obtained from a measurement on a liquid phantom confirms that the optimal time gates obtained through our simulation method can be used for optimizing TD-DCS monitoring of CBF on patients.

## Code, Data, and Materials Availability

Data supporting the results reported in the paper can be requested by contacting the corresponding author.
