# Supplementary material for: Optimization of time domain diffuse correlation spectroscopy parameters for measuring brain blood flow
Source: Neurophotonics. 2021 Aug 12;8(3):035005. doi: 10.1117/1.NPh.8.3.035005 (PMC8358828; doi:10.1117/1.NPh.8.3.035005)
Supplement: Supplementary file 1 [file NPh_008_035005_SD001.pdf]

Optimization of time domain diffuse correlation spectroscopy (TD-DCS) parameters for measuring brain blood flow

Dibbyan Mazumder, Melissa M. Wu, Nisan Ozana, Davide Tamborini, Maria Angela Franceschini, Stefan A. Carp

Supplementary material

We have compared the performance of different Gaussian IRFs with varying FWHMs to determine the “ideal” Gaussian FWHM. Figures S1 and S2 show the intrinsic sensitivities across time gates for various time Gaussian IRFs at 765 and 1064 nm, respectively. The CNR comparison has been shown in Figs. S3 and S4 for 765 and 1064 nm excitation wavelengths, respectively. The superficial sensitivities at 765 and 1064 nm wavelengths for various Gaussian IRFs have been shown in Figs. S5 and S6, respectively. Finally, the figure of merit (FoM) across time gates for the Gaussian IRFs are shown in Figs. S7 and S8 for 765 and 1064 nm, respectively. Here, we did not include any Gaussian IRF with FWHM value greater than 450 ps because as we can see in Figs. S1 and S2, the intrinsic sensitivities at the later time gates start to decrease with increase in the width of the IRF. On the other hand, we did not include Gaussian IRFs with FWHM less than 200 ps as CNR decreases with decrease in laser pulse width (Figs. S3 and S4) due to increase in noise, introduced due to lack of sufficient coherent length of the source.

Figure S7 shows that for 765 nm, the highest FoM can be achieved by Gaussian shaped IRFs with FWHMs between 300 to 400 ps for a gate start delay of 400 ps and width above 700 ps. Figure S8 shows the highest FoM for 1064 nm is seen for a 300 ps FWHM, for time gates with gate start time of 600 ps from the peak of the TPSF and widths above 700 ps. Hence, here in this paper, we have considered the Gaussian IRF with FWHM of 300 ps as the “ideal” reference IRF.

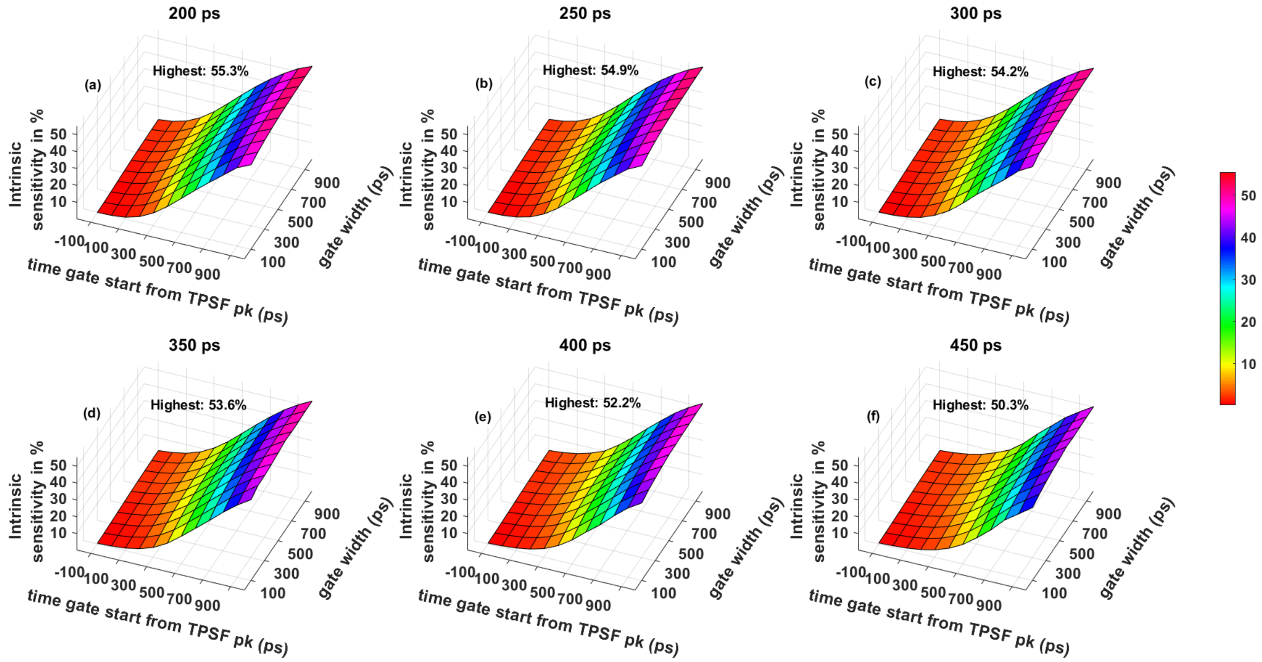

Fig. S1: Intrinsic sensitivities across various time gates at 765 nm for Gaussian IRFs of varying FWHMs: (a) 200 ps, (b) 250 ps, (c) 300 ps, (d) 350 ps, (e) 400 ps and (f) 450 ps.

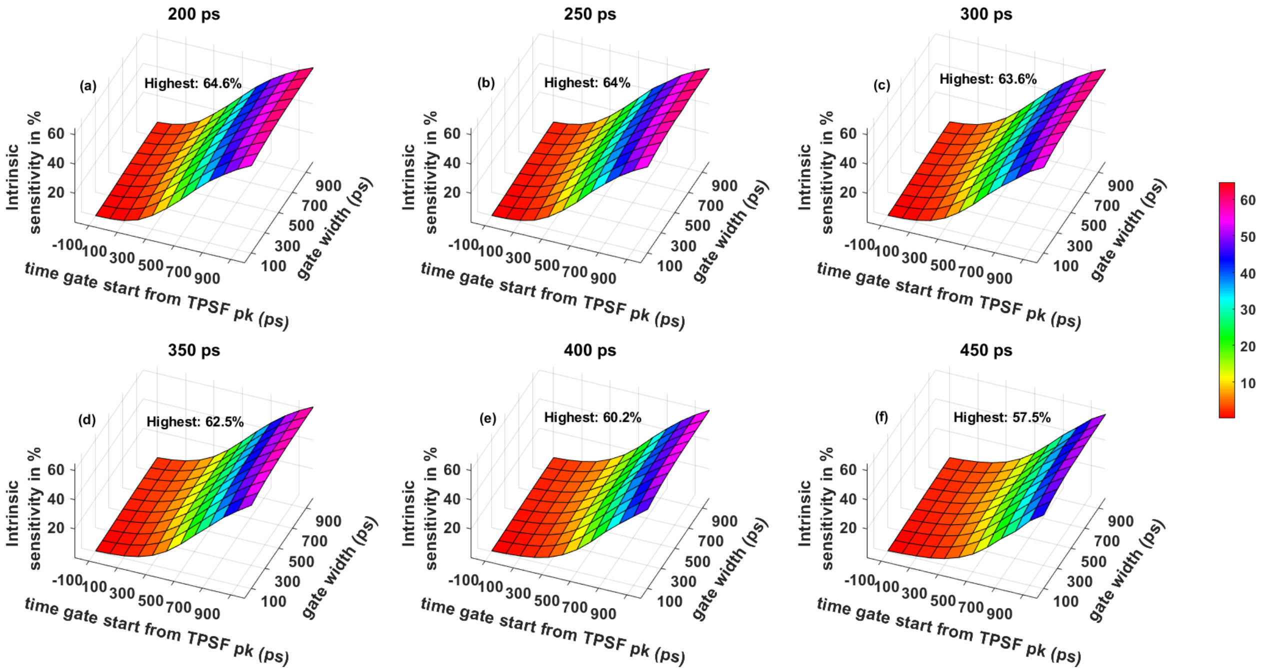

Fig. S2: Intrinsic sensitivities across various time gates at 1064 nm for Gaussian IRFs of varying FWHMs: (a) 200 ps, (b) 250 ps, (c) 300 ps, (d) 350 ps, (e) 400 ps and (f) 450 ps.

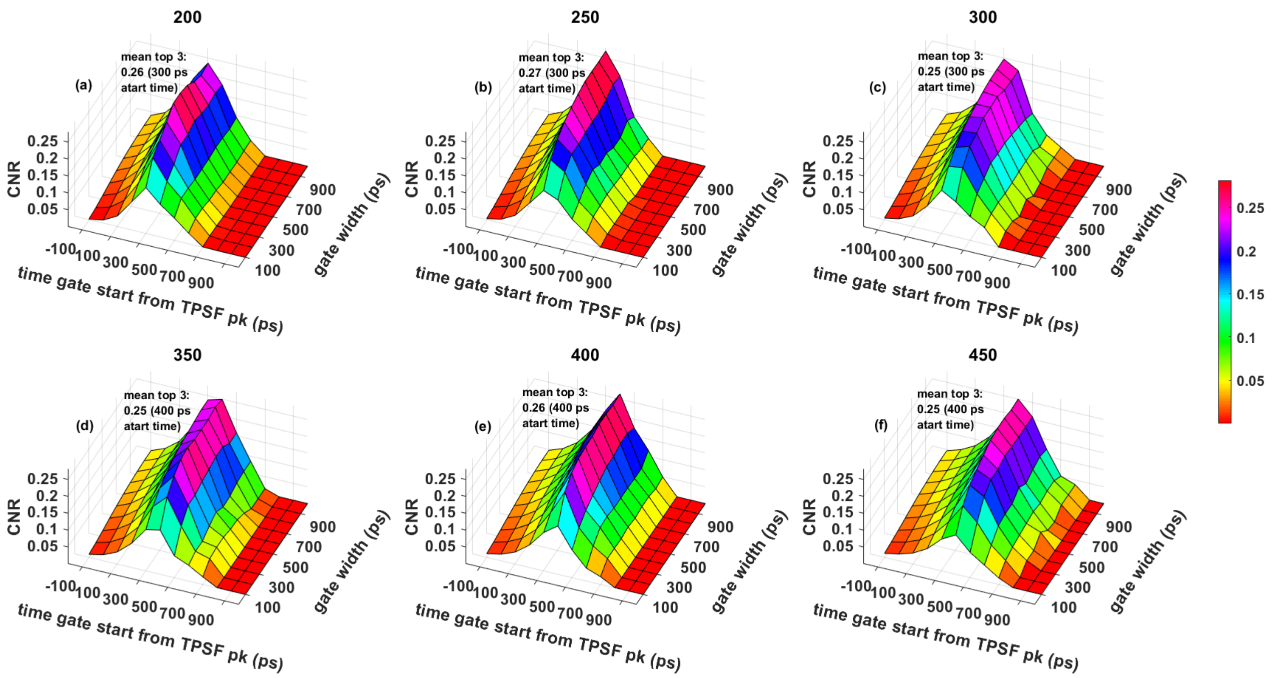

Fig. S3: Contrast-to-noise ratios (CNR) across various time gates at 765 nm for Gaussian IRFs of varying FWHMs: (a) 200 ps, (b) 250 ps, (c) 300 ps, (d) 350 ps, (e) 400 ps and (f) 450 ps.

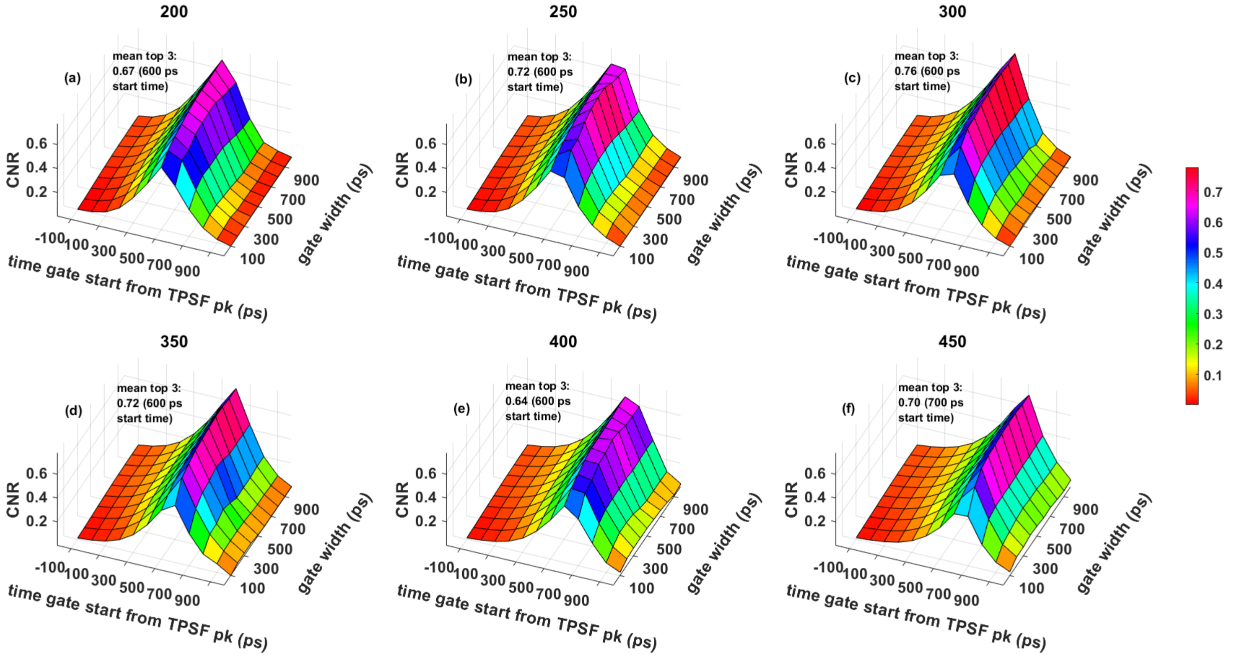

Fig. S4: Contrast-to-noise ratios (CNR) across various time gates at 1064 nm for Gaussian IRFs of varying FWHMs: (a) 200 ps, (b) 250 ps, (c) 300 ps, (d) 350 ps, (e) 400 ps and (f) 450 ps.

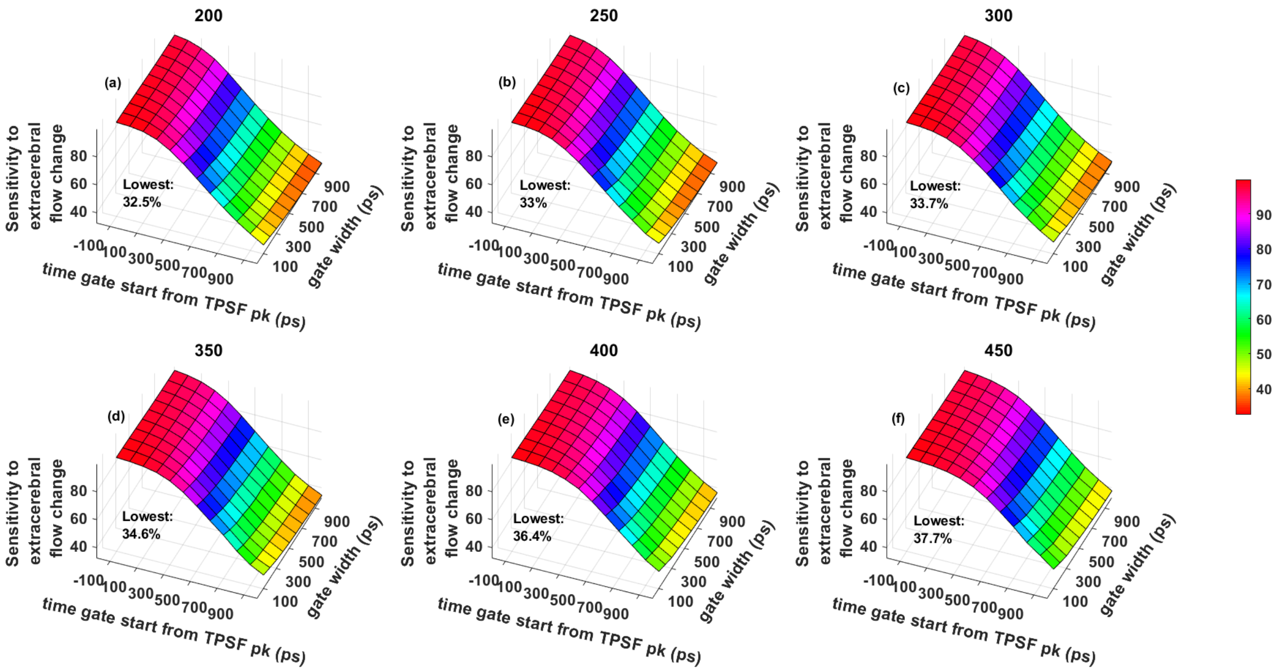

Fig. S5: Superficial sensitivities across various time gates at 765 nm for Gaussian IRFs of varying FWHMs: (a) 200 ps, (b) 250 ps, (c) 300 ps, (d) 350 ps, (e) 400 ps and (f) 450 ps.

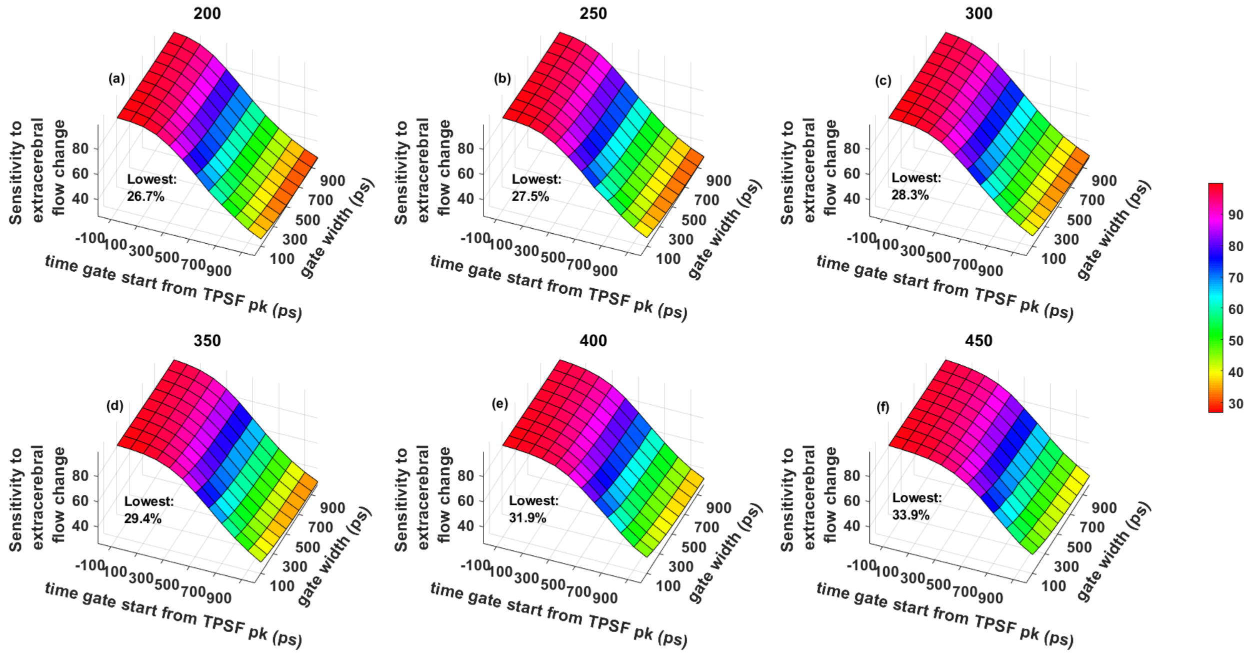

Fig. S6: Superficial sensitivities across various time gates at 1064 nm for Gaussian IRFs of varying FWHMs: (a) 200 ps, (b) 250 ps, (c) 300 ps, (d) 350 ps, (e) 400 ps and (f) 450 ps.

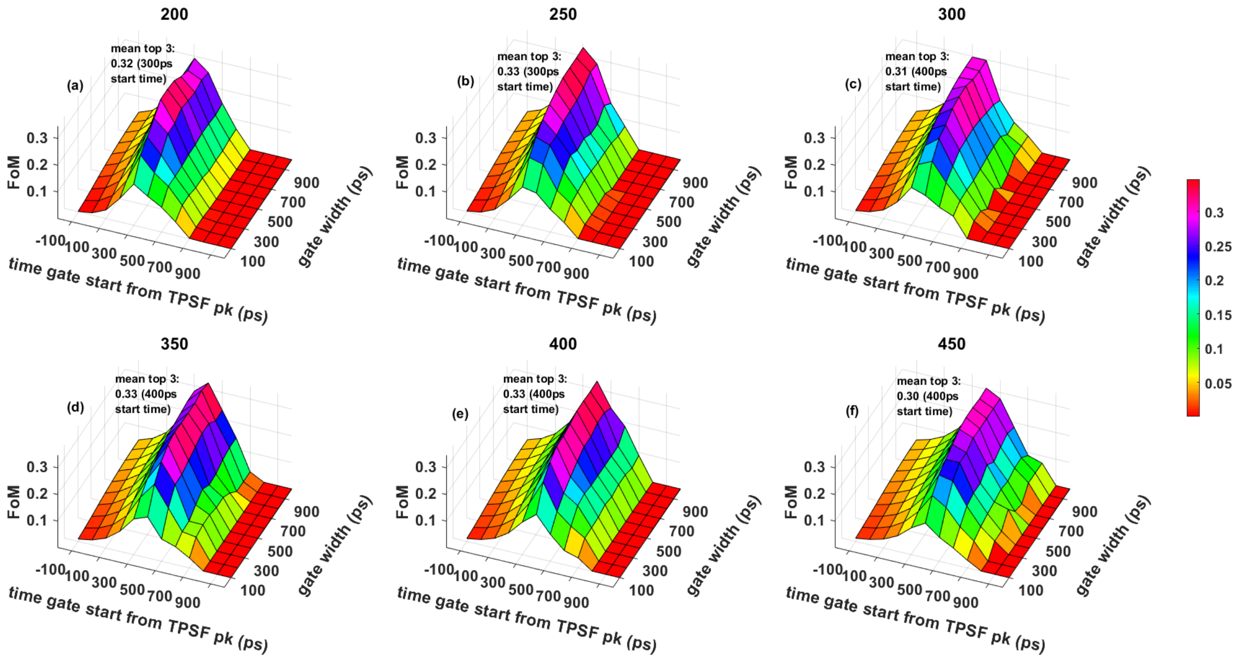

Fig. S7: Figure of merit (FoM) across various time gates at 765 nm for Gaussian IRFs of varying FWHMs: (a) 200 ps, (b) 250 ps, (c) 300 ps, (d) 350 ps, (e) 400 ps and (f) 450 ps.

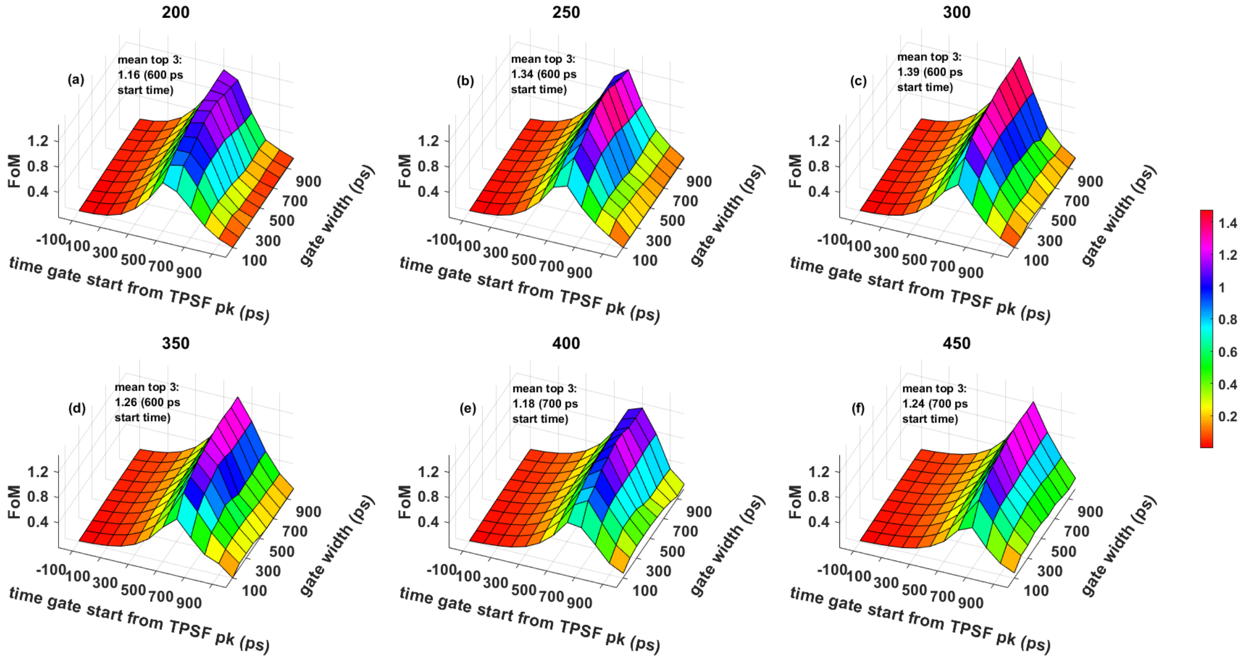

Fig. S8: Figure of merit (FoM) across various time gates at 1064 nm for Gaussian IRFs of varying FWHMs: (a) 200 ps, (b) 250 ps, (c) 300 ps, (d) 350 ps, (e) 400 ps and (f) 450 ps.

The table showing the time difference between the occurrence of the peak of the instrument response function (IRF) and peak of the temporal point spread function (TPSF) has been shown in Table S1.

Table S1 : Time duration between the IRF peak and TPSF peak for different source-detector combinations

IRF	Time duration between IRF peak and TPSF peak in ps
Gaussian IRF – 765 nm	190
VisIR-FG– 765 nm	270
VisIR-FG with EOM– 765 nm	190
VisIR-RE– 765 nm	290
VisIR-RE with EOM– 765 nm	180
Gaussian IRF – 1064 nm	180
1064-NW-1064 nm	200
1064-NW with EOM -1064 nm	160
